# Comprehensive Pan-Cancer Analysis of Senescence With Cancer Prognosis and Immunotherapy

**DOI:** 10.3389/fmolb.2022.919274

**Published:** 2022-07-15

**Authors:** Qinfei Zhao, Weiquan Hu, Jing Xu, Shaoying Zeng, Xuxiang Xi, Jing Chen, Xiangsheng Wu, Suping Hu, Tianyu Zhong

**Affiliations:** ^1^ Department of Laboratory Medicine, First Affiliated Hospital of Gannan Medical University, Ganzhou, China; ^2^ Department of Joint Surgery, Ganzhou People’s Hospital, Ganzhou, China; ^3^ Department of Orthopaedic Surgery, Sun Yat-Sen Memorial Hospital, Sun Yat-Sen University, Guangzhou, China; ^4^ Department of Obstetrics and Gynecology, First Affiliated Hospital of Gannan Medical University, Ganzhou, China; ^5^ Department of Emergency, First Affiliated Hospital of Gannan Medical University, Ganzhou, China

**Keywords:** senescence, pan-cancer, prognosis, tumor-immune microenvironment, immunotherapy

## Abstract

Senescence is a double-edged sword in tumorigenesis and affects the immunotherapy response through the modulation of the host’s immune system. However, there is currently a lack of comprehensive analysis of the senescence-related genes (SRGs) in human cancers, and the predictive role of senescence in cancer immunotherapy response has not been explored. The multi-omics approaches were performed in this article to conduct a systematic pan-cancer genomic analysis of SRGs in cancer. In addition, we calculated the generic senescence score (SS) to quantify the senescence levels in cancers and explored the correlations of SS with cancer prognosis, biological processes, and tumor microenvironment (TME). The gene signatures were deregulated in multiple cancers and indicated a context-dependent correlation with prognosis, tumor-immune evasion, and response to therapy across various tumor types. Further analysis disclosed that SS was positively associated with the infiltration levels of immune suppressive cells, including induced Tregs (iTregs), central memory Ts (Tcms), and natural Tregs (nTregs), and negatively associated with immune killer cells, including natural killers (NKs) and mucosal-associated invariant Ts (MAITs). Moreover, the SS was significantly correlated with tumor-associated macrophages (TAMs), cancer-associated fibroblasts (CAFs), immune-related genes, and immune checkpoints and had a predictive value of immunotherapy response. Thus, the expression of SRGs was involved in resistance to several anticancer drugs. Our work illustrates the characterization of senescence across various malignancies and highlights the potential of senescence as a biomarker of the response to immunotherapy.

## Introduction

Despite considerable progress in understanding cancer’s genetic and immunological underpinnings, cancer remains a major and ever-growing health burden globally (Siegel et al., 2021). Nevertheless, increasing evidence has suggested that senescence and the tumor microenvironment (TME) exert crucial roles in cancer initiation, progression, metastasis, recurrence, and response to therapy (Fane and Weeraratna, 2020). Senescence exerts complex effects on the innate and adaptive immune systems, a process termed immunosenescence (Xu et al., 2017; Pereira et al., 2019). Immune cell senescence occurs largely in T cells. Other immune effector cell types crucial for tumor immunity are associated with decreasing immune surveillance and are a strong risk factor for cancer (Li et al., 2019; Pereira et al., 2019; Fane and Weeraratna, 2020). While senescence can impact many immune cell subsets, the mechanisms adopted are still unknown.

Contrary to the preconceived notion that the processes of decreased function and fitness of senescence oppose the processes of hyperproliferation and increased cellular survival of cancer in the context of a cell, studies highlight that several hallmarks of senescence are shared with cancer (Aunan et al., 2017). Recent studies demonstrate that senescent cells are characterized by the acquisition of inflammatory phenotype, defined as senescence-associated secretory phenotype, whereby cells produce and secrete pro-inflammatory cytokines, chemokines, growth factors, and proteases and, in turn, regulate the microenvironment and cell growth (Krtolica et al., 2001; Lawrenson et al., 2010; Kim et al., 2013). In addition, aberrant expression of the senescence-related gene (SRG) has been widely reported in various cancers (Testoni et al., 2015; Wang et al., 2016; Landa et al., 2019; Huang et al., 2020; Toufektchan et al., 2020). Mutations of some SRGs have been considered cancer drivers (Iacobucci et al., 2016; Giacomelli et al., 2018; Connor et al., 2019). For example, the TP53 gene was the most frequently mutated in human cancers, including colorectal and lung cancer (Rudin et al., 2009; Alam et al., 2016). Missense mutations in TP53 and deletion mutations in RB1 were the most frequent mutations in small cell lung cancer (Augert et al., 2017). The simultaneous deletion of CDKN2A and CDKN2B promotes tumorigenesis in pancreatic cancer (Tu et al., 2018).

The TME comprises infiltrating immune cells, tumor-associated fibroblasts, endothelial cells, and extracellular components (Balkwill et al., 2012; Liu et al., 2017). Cancer-associated fibroblasts (CAFs), tumor-associated macrophages (TAMs), regulatory T cells (Tregs), and dendritic cells (DCs) are essential components of the inhibitory cancer microenvironment that interact with tumor cells in the TME to drive the therapeutic resistance and malignant phenotype of tumor cells, such as proliferation, invasion and metastasis, and therapeutic resistance (Labidi-Galy et al., 2012; Albini et al., 2018; Munn et al., 2018; Sun et al., 2018; Lawal et al., 2021). In addition, emerging evidence has shown that the accumulation of senescent stromal cells contributes strongly to generating a tumor-permissive, chronic inflammatory microenvironment to shelter incipient tumor cells, thus allowing them to grow and progress unaffected by the immune system (Ruhland et al., 2016; Fane and Weeraratna, 2020). However, investigating the role of senescence in TME is still in its infancy and needs further exploration and validation.

Here, a systematic pan-cancer analysis to elucidate the potential impact of comprehensive integrative multi-omics analysis of SRGs in cancers was performed. Subsequently, the correlations between senescence score (SS) and survival, biological pathways, and immune features were explored. Our findings highlight the importance of senescence across cancers and offer a framework for new cancer therapeutics.

## Materials and Methods

### Data Collection

The gene list of senescence was obtained from http://www.gsea-msigdb.org/gsea/index.jsp, and the complete listing of 33 SRGs is presented in Supplementary Table S1. Gene expression data and clinical information on cancer and corresponding normal tissues were derived from the Cancer Genome Atlas (TCGA) and the Genotype-Tissue Expression (GTEx) through the tool University of California Santa Cruz (UCSC) Xena (https://xena.ucsc.edu/). The TCGA cancer types are listed in Supplementary Table S2. The complete microarray and clinical datasets of bladder cancer (BC) [GSE13507 (Lee et al., 2010)], urothelial cancer (UC) [GSE32894 (Sjödahl et al., 2012)], and nonsmall cell lung cancer (NSCLC) [GSE61676 (Baty et al., 2017)] were retrieved from the Gene Expression Omnibus database of the National Center for Biotechnology Information database (https://www.ncbi.nlm.nih.gov/). The gene expression data of the UC dataset were retrieved from http://research-pub.gene.com/IMvigor210CoreBiologies (Mariathasan et al., 2018).

### Evaluation of Senescence Score

To investigate the senescence level in a tumor, we calculated the generic SS using the method of single-sample gene-set enrichment analysis (ssGSEA) in the R gene-set variation analysis (GSVA) package (Hänzelmann et al., 2013) and used the senescence gene set to quantify the gene expression levels for each tumor type. We evaluated the SS between cancerous and normal samples in 33 tumors from TCGA. We defined a tumor as SS-high if its SS was in the upper half of all SS in the same tumor type, and as SS-low if its SS was in the lower half.

### Differentially Expressed Gene Analysis

To determine the expression difference between tumor and normal samples, we conducted the differential expression analysis of SRGs from 31 cancers, utilizing the R “limma” package (Ritchie et al., 2015). Genes with |Log2-fold change (FC)| greater than one and p-values less than 0.05 were regarded as significantly differentially expressed.

### Construction of Protein-Protein Interaction Network

The Search Tool for the Retrieval of Interacting Genes (STRING) database (https://string-db.org/) was used to construct a protein-protein interaction (PPI) network based on the SRGs. The threshold value required confidence (combined score) >0.4.

### Survival Analysis

We utilized the “survminer” and “survival” R packages to examine cancer patients’ survival prognoses, including overall survival (OS), disease-specific survival (DSS), progression-free interval (PFI), disease-free interval (DFI), and progression-free survival (PFS). The *p*-values were calculated using the log-rank test.

### Genetic Alteration Analysis

The website cBio Cancer Genomics Portal (cBioPortal) was utilized to investigate the genomic cancer alterations for a specific gene (www.cbioportal.org) (Cerami et al., 2012). We applied the R “ggplot2” package to visualize genomic alterations of SRGs among 32 tumor types of TCGA.

### Single Nucleotide Variation Analysis

We obtained the single nucleotide variation (SNV) data of 10,234 samples and evaluated the frequencies and clinical effects of seven variant types of deleterious mutations of SRGs across 33 different tumors from the TCGA database. We filtered out the Silent, Intron, IGR, 3′ UTR, 5′ UTR, 3′ Flank, and 5′ Flank to calculate the SNV percentage. Then, the SNV mutation percentage of each gene’s coding region was calculated using the number of mutated samples/number of cancer samples (Liu et al., 2018). Thus, we employed maftools to visualize and summarize the SNV data (Mayakonda et al., 2018).

### Copy Number Variation Analysis

To identify regions with significantly altered amplification or deletion across sets of patients, copy number variation (CNV) data (*n* = 11,495) of 33 cancer types were obtained from the TCGA database and processed by Genomic Identification of Significant Targets in Cancer Scores (GISTICS) 2.0. We employed GISTIC-processed CNV data to perform the percentage statistics based on CNV subtypes and calculated the relationship between CNV and mRNA expression utilizing raw CNV data and RNA-seq by expected maximization normalized mRNA expression data. Only genes presenting >5% CNV in cancers were considered. The correlation between mRNA expression and the CNV percentage of samples was computed using a Person’s product-moment correlation coefficient, as described by Schlattl et al. (2011).

### Methylation Analysis

DNA methylation data of 10,129 samples were derived from the TCGA database. Fourteen cancer types with more than 10 paired tumor/adjacent nontumor samples were selected and processed for differential methylation analysis. The student’s *t*-test was used to assess the methylation difference between cancerous and normal samples. In general, there is more than one methylation site in the region of each gene. As a result, numerous tags store the methylation level per site. We conducted the correlation analysis for methylation and mRNA expression to filter the site most negatively correlated with gene expression in this module.

### Gene-Set Variation Analysis

We conducted GSVA (Hänzelmann et al., 2013) on all TCGA samples utilizing the 50 Hallmark Pathways from the molecular signature database (MSigDB) to gain further insights into biological implications. First, the pathway activity score was computed by ssGSEA (Macosko et al., 2015) for overall tumors, and then Spearman correlations with SS were calculated.

### Immune Feature Analysis

We adopted the Estimation of STromal and Immune cells in MAlignant Tumor tissues using the Expression data (ESTIMATE) algorithm (Yoshihara et al., 2013) to compute the stromal score, immune score, estimate score, and tumor purity. Besides, we used the method of Zeng et al. (2019) to evaluate the immune-related and other biological processes in TME. The relationship between SS and immune cell infiltration was determined using two databases, namely Immune Cell Abundance Identifier (ImmuCellAI) (http://bioinfo.life.hust.edu.cn/ImmuCellAI) and Tumor Immune Estimation Resource 2.0 (TIMER2.0) (http://timer.cistrome.org) databases. The Cell-type Identification by Estimating Relative Subsets of RNA Transcripts (CIBERSORT) algorithm (Newman et al., 2015) was utilized to assess the relative fractions of 22 infiltrating immune cell types for each of the SRGs in each tumor.

### Tumor Mutation Burden and Microsatellite Instability

TMB, defined as the total number of mutations per coding area of a tumor genome in specific cancer, has been reported to be closely associated with the effectiveness of cancer immunotherapy. The TCGA pan-cancer mutation data were obtained from the UCSC Xena database, and the TMB score was calculated. MSI is a tumor phenotype for strong responses to immunotherapy and is usually caused by a deficiency of the DNA mismatch repair (MMR) system that leads to genomic instability. The MSI data were downloaded from a recent study (Bonneville et al., 2017). Thus, the relationship of SS with TMB or MSI was examined using Spearman’s correlation coefficient.

### Drug Sensitivity Analysis

We collected the SRGs’ expression and drug sensitivity data from the Genomics of Drug Sensitivity in Cancer (GDSC) and the Cancer Therapeutics Response Portal (CTRP) projects. Pearson’s correlation analysis was carried out to evaluate the correlation between SRG expression and half maximal inhibitory concentration 50 (IC50) values of drugs.

### Statistical Analysis

Correlation analyses between two variables were conducted with the Spearman correlation test unless otherwise specified. The difference between groups was determined by utilizing a student’s *t*-test. The data were presented as means ± standard error (SD). A Cox proportional hazards model was used to calculate survival risk and hazard ratio (HR). A *p*-value < 0.05 was considered statistically significant. All statistical analyses were carried out using R software (version 4.0.2).

## Results

### Senescence-Related Genes are Deregulated in Tumors, With Their Expression Varying Substantially Across 33 Tumor Types

**FIGURE 1:**
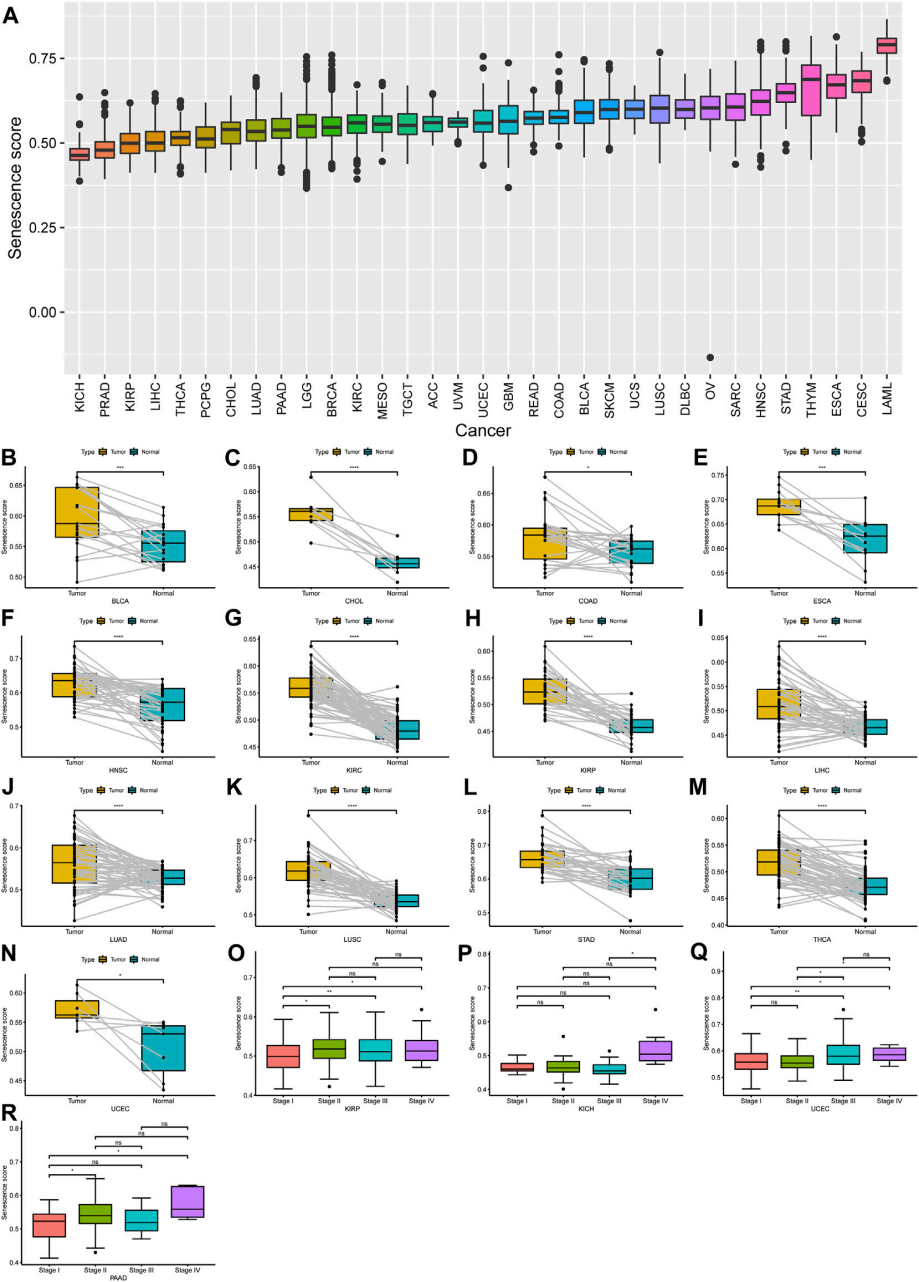
Expression landscape of senescence-related genes (SRGs) in human cancer. **(A)** Spectra of senescence score (SS) across 33 different cancer types; 33 tumor types were ordered according to increasing SS from left to right. **(B–N)** A significant difference of SS was observed in 13 tumor types. **(O–R)** The SS was analyzed by the main pathological stages (stage I, stage II, stage III, and stage IV) in kidney renal papillary cell carcinoma, kidney chromophobe, uterine corpus endometrial carcinoma, and pancreatic adenocarcinoma. (*p* < 0.05 was considered significant, *p < 0.05, **p < 0.01, ***p < 0.001, and ****p < 0.0001).

To further understand the underlying biological mechanisms of senescence in cancers, we performed the ssGSEA to compute the SS of 33 tumors. The results demonstrated that the SS differed widely in different tumor types (Figure 1A). To note, acute myeloid leukemia (LAML) had the highest senescence level, whereas kidney chromophobe (KICH) had the lowest (Figure 1A). Furthermore, compared with paired normal tissues, out of these 33 cancers, we observed that the SS was significantly increased in 13 cancers, including urothelial bladder carcinoma (BLCA), cholangiocarcinoma (CHOL), colon adenocarcinoma (COAD), esophageal carcinoma (ESCA), head and neck squamous cell carcinoma (HNSC), kidney renal clear cell carcinoma (KIRC), kidney renal papillary cell carcinoma (KIRP), liver hepatocellular carcinoma (LIHC), lung adenocarcinoma (LUAD), lung squamous cell carcinoma (LUSC), stomach adenocarcinoma (STAD), thyroid carcinoma (THCA), and uterine corpus endometrial carcinoma (UCEC), while there was no significant difference for other cancers (Figures 1B–N). Subsequently, we analyzed the relationship of SS with the pathological tumor stages. We observed a significant increase in SS with increasing tumor stage in KIRP, KICH, UCEC, and pancreatic adenocarcinoma (PAAD) (Figures 1O–R), but not in others.

**FIGURE 2:**
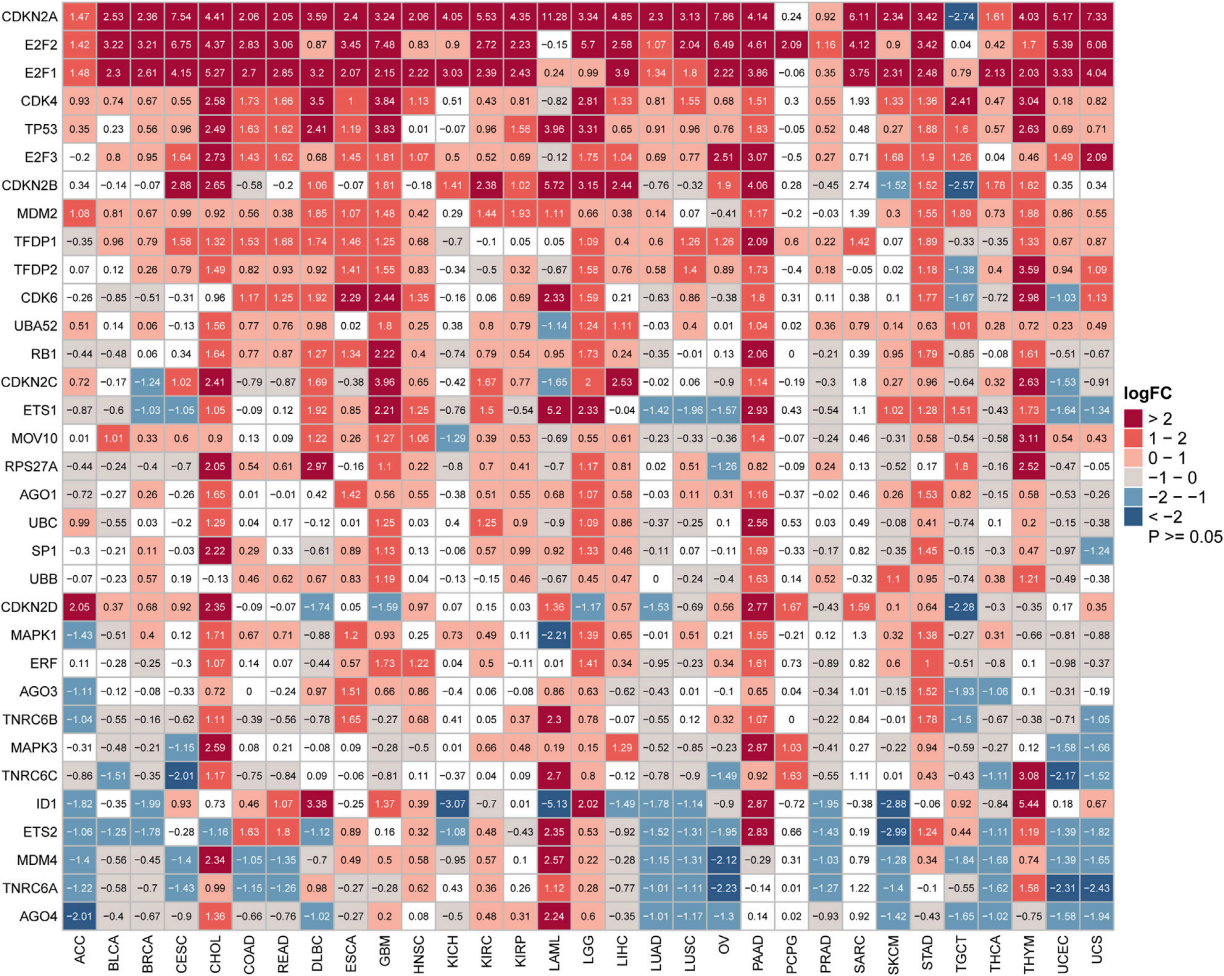
Aberrant expression of senescence-related genes in cancers. Heatmap showing the mRNA difference of senescence gene set between the Cancer Genome Atlas (TCGA) tumor samples and normal tissue samples from TCGA and Genotype-Tissue Expression. Upregulated genes have logFC > 0, and downregulated genes have logFC < 0. (*p* < 0.05 was considered to be significant. FC: fold change).

Furthermore, we further evaluated the differential expression of 33 SRGs among cancers from the GTEx and TCGA pan-cancer databases. The distribution of the 33 SRGs’ expression levels in these 33 cancers is shown in Figure 2. Our results suggested that all SRGs were abnormally expressed in various cancers with respect to normal tissues (Figure 2). In addition, several SRGs showed consistent patterns for expression across different cancers. For example, CDKN2A, E2F1, E2F2, and CDK4 were significantly overexpressed in 28, 26, 24, and 15 cancers, respectively (Figure 2). Interestingly, some SRGs exhibited cancer-type-specific expression patterns. For example, CDKN2A was highly upregulated for almost all cancer types but apparently suppressed in testicular germ cell tumors (TGCT) (log2FC = −2.74). Thus, the PPI network based on the 33 SRGs was constructed in the STRING database (Supplementary Figure S1). These findings showed that the dysregulated expression of the SRGs might have interesting, distinctive correlations with different cancers in tumorigenesis.

### Senescence-Related Gene Expression is Significantly Associated With Patient Prognosis in Many Tumor Types

**FIGURE 3:**
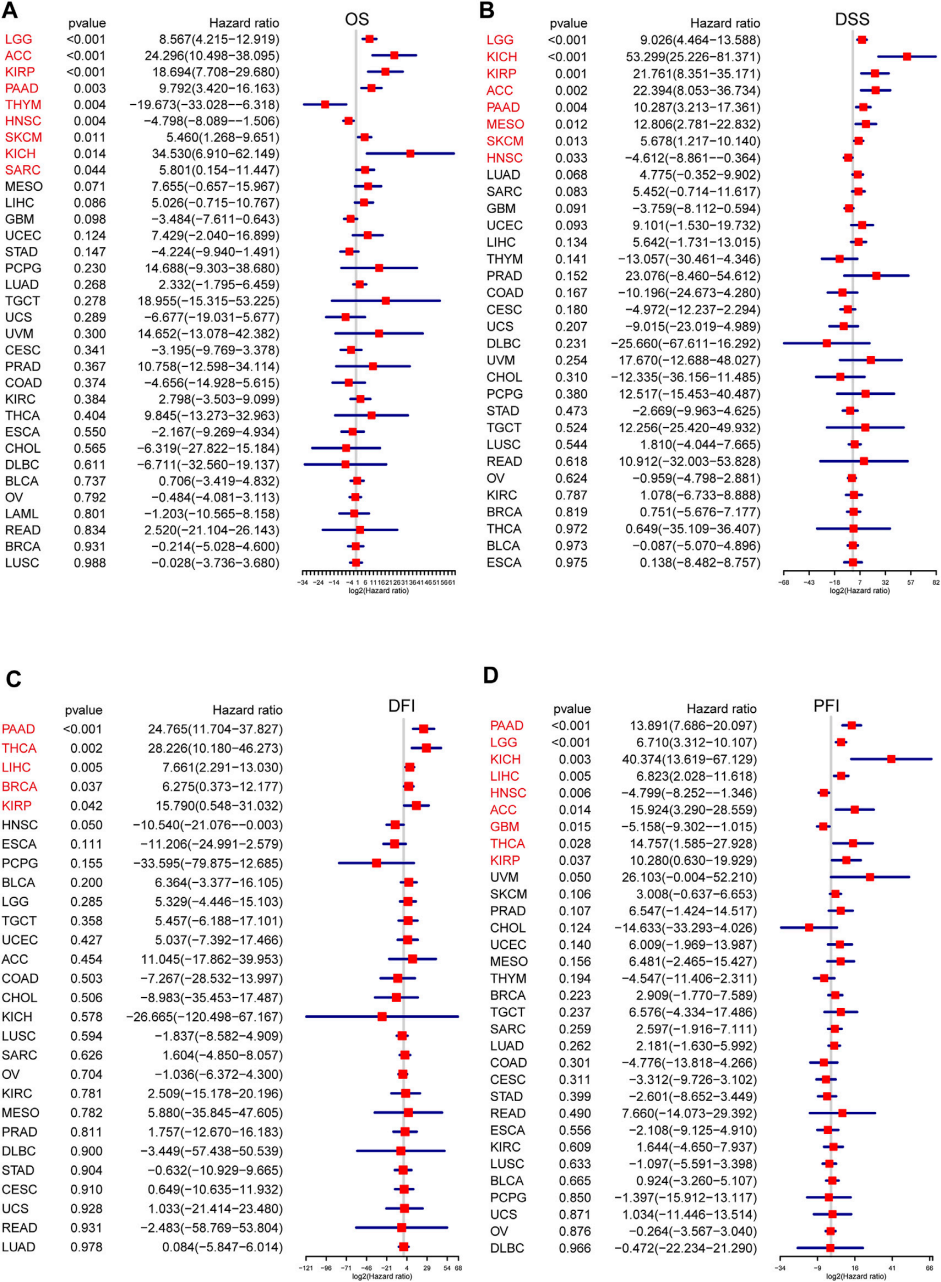
The prognosis value of senescence score (SS) in each cancer type. The association of SS with prognosis in pan-cancer was evaluated based on Cox regression. **(A)** Correlations of SS with overall survival in each cancer. **(B)** Correlations of SS with disease-specific survival in each cancer. **(C)** Correlations of SS with disease-free interval in each cancer. **(D)** Correlations of SS with progression-free interval in each cancer. Hazard ratio (HR) above 1 indicates an adverse prognosis, and the HR value less than 1.0 indicates a good prognosis. Tumors with significant *p*-values were shown in red font, and X-axis represents log2(HR). (Spearman correlation, *p* < 0.05 was considered significant).

Next, to further examine the relationship between the senescence levels and survival outcomes of patients, survival analysis was executed by univariate Cox regression. Generally, the associations between SS and the cancer patients’ prognosis were consistent in OS, DSS, DFI, and PFI. However, compared with the OS, DSS, and PFI, SS in only a few tumors was associated with DFI. To note, SS was clearly correlated with OS in nine types of cancer, including lower grade glioma (LGG), adrenocortical carcinoma (ACC), KIRP, PAAD, thymoma (THYM), HNSC, skin cutaneous melanoma (SKCM), KICH, and sarcoma (SARC) (Figure 3A). Specifically, SS seemed to be a significant risk factor in seven cancer types: LGG (*p* < 0.001, HR = 8.567), ACC (*p* < 0.001, HR = 24.296), KIRP (*p* < 0.001, HR = 18.694), PAAD (*p* = 0.003, HR = 9.792), SKCM (*p* = 0.011, HR = 5.460), KICH (*p* = 0.014, HR = 34.530), and SARC (*p* = 0.044, HR = 5.801; Figure 3A). In addition, SS was a protective factor in two other types of cancer: THYM (*p* = 0.004, HR = −19.673) and HNSC (*p* = 0.004, HR = −4.798; Figure 3A). Besides, SS affected patients’ DSS in eight cancer types, including LGG, KICH, KIRP, ACC, PAAD, Mesothelioma (MESO), SKCM, and HNSC (Figure 3B). In particular, SS was more likely to have a harmful effect in LGG (*p* < 0.001, HR = 9.026), KICH (*p* < 0.001, HR = 53.299), KIRP (*p* = 0.001, HR = 21.761), ACC (*p* = 0.002, HR = 22.394), PAAD (*p* = 0.004, HR = 10.287), MESO (*p* = 0.012, HR = 12.806), and SKCM (*p* = 0.013, HR = 5.678) and conversely in HNSC (*p* = 0.033, HR = −4.612; Figure 3B). Subsequently, we assessed the relationship between SS and DFI. We observed that the elevated SS was correlated with poor prognosis in PAAD (*p* < 0.001, HR = 24.765), THCA (*p* = 0.002 HR = 28.226), LIHC (*p* = 0.005, HR = 7.661), Breast invasive carcinoma (BRCA) (*p* = 0.037, HR = 6.275), and KIRP (*p* = 0.042, HR = 15.790; Figure 3C). Moreover, we examined the relationship between SS and PFI and found that high SS impacted PFI unfavorably in PAAD (*p* < 0.001, HR = 13.891), LGG (*p* < 0.001, HR = 6.710), KICH (*p* = 0.003, HR = 40.374), LIHC (*p* = 0.005, HR = 6.823), ACC (*p* = 0.014, HR = 15.924), THCA (*p* = 0.028, HR = 14.757), and KIRP (*p* = 0.037, HR = 10.280), but favorably in HNSC (*p* = 0.006, HR = −4.799) and Glioblastoma multiforme (GBM) (*p* = 0.015, HR = −5.158; Figure 3D). In conclusion, these results demonstrated that SS was correlated significantly with the prognoses in cancer patients, especially those with PAAD and KIRP.

**FIGURE 4:**
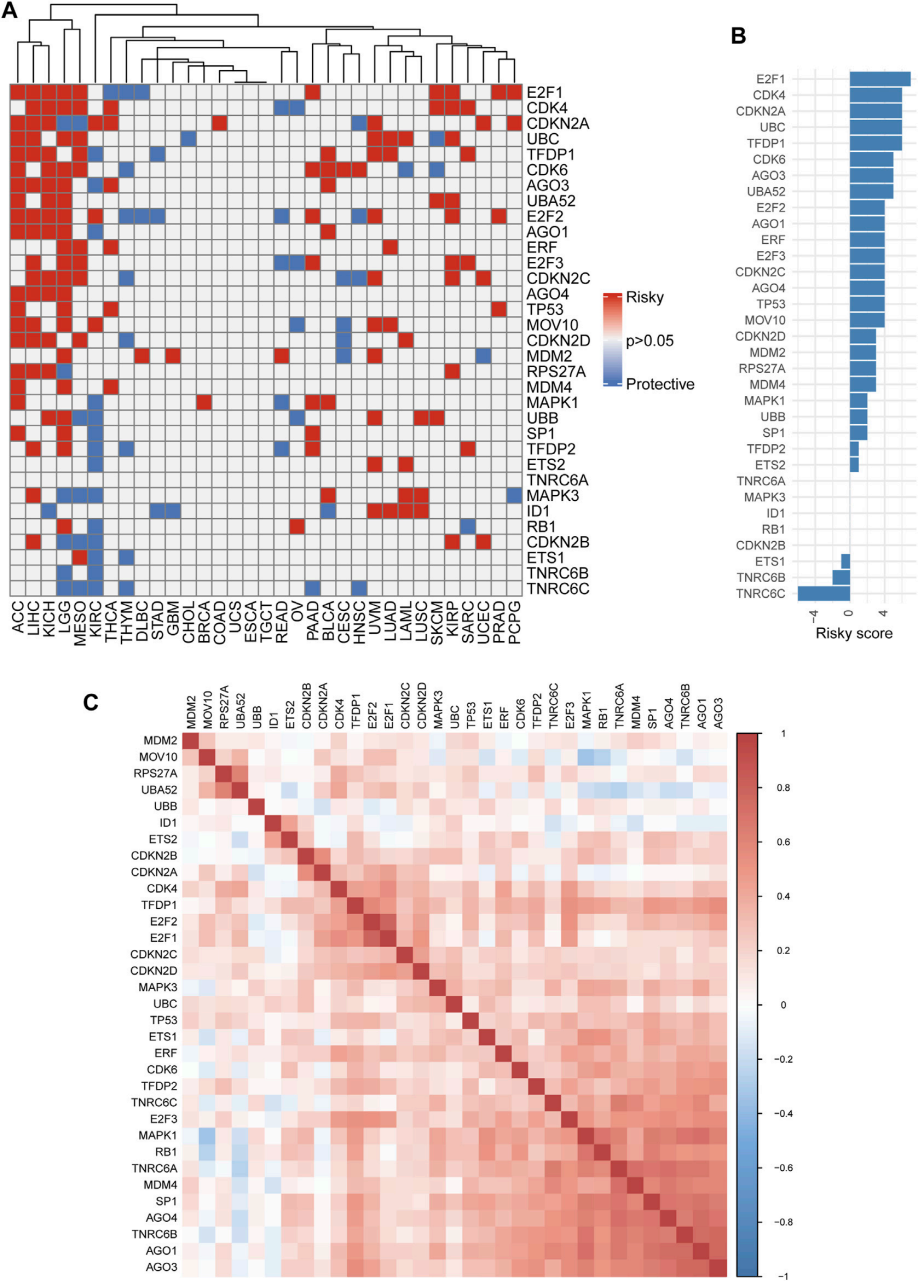
Single nucleotide variation (Survival analysis of senescence-related genes (SRGs). **(A)** Heatmap showing univariate Cox regression analysis of SRGs (*p* > 0.05: all gray; *p* < 0.05: hazard ratio (HR) > 1 indicates red and HR < 1 indicates blue). **(B)** The summary statistics for Cox regression analysis of all 33 senescence genes across 33 tumors. Senescence genes were sorted by descending number of a risky score (for risk factor, +1; for protective factor, −1). **(C)** The correlation plot determined with the Spearman correlation test results show the correlation of gene expression among SRGs across 33 cancer types. A positive correlation is indicated in red, and a negative correlation is denoted in blue. A darker color indicates stronger correlations (Spearman correlation, *p* < 0.05 was considered significant).

We also assessed the prognostic effect of SRGs with clinical relevance in 33 cancers. As a whole, the ETS1, TNRC6B, and TNRC6C genes were thought to be preventive against tumors, while the remaining SRGs appeared linked to cancer risk (Figures 4A,B). Some genes, however, showed discrepant risk patterns. By way of example, AGO3 could increase the risk of multiple types of cancer while playing a protective role in only KIRC. Similar results were also observed in AGO1, RPS27A, SP1, and ETS2 of several cancers. On the contrary, ETS1 was a protective gene in KIRC and THYM but a risk gene in MESO. The correlation analyses among the members of SRGs revealed an overall positive correlation with each other, which was meaningful for us to understand the SRGs’ mode of action (Figure 4C). Also, according to the expression and prognosis of pan-cancer for each of the SRGs (Figures 2, 4A), a summary table is given in Supplementary Table S3, which reveals the potential biomarkers of certain human cancers. Taken together, the SRGs exhibited heterogeneous prognoses across different cancer types.

### Genetic Alteration of Senescence-Related Genes

**FIGURE 5:**
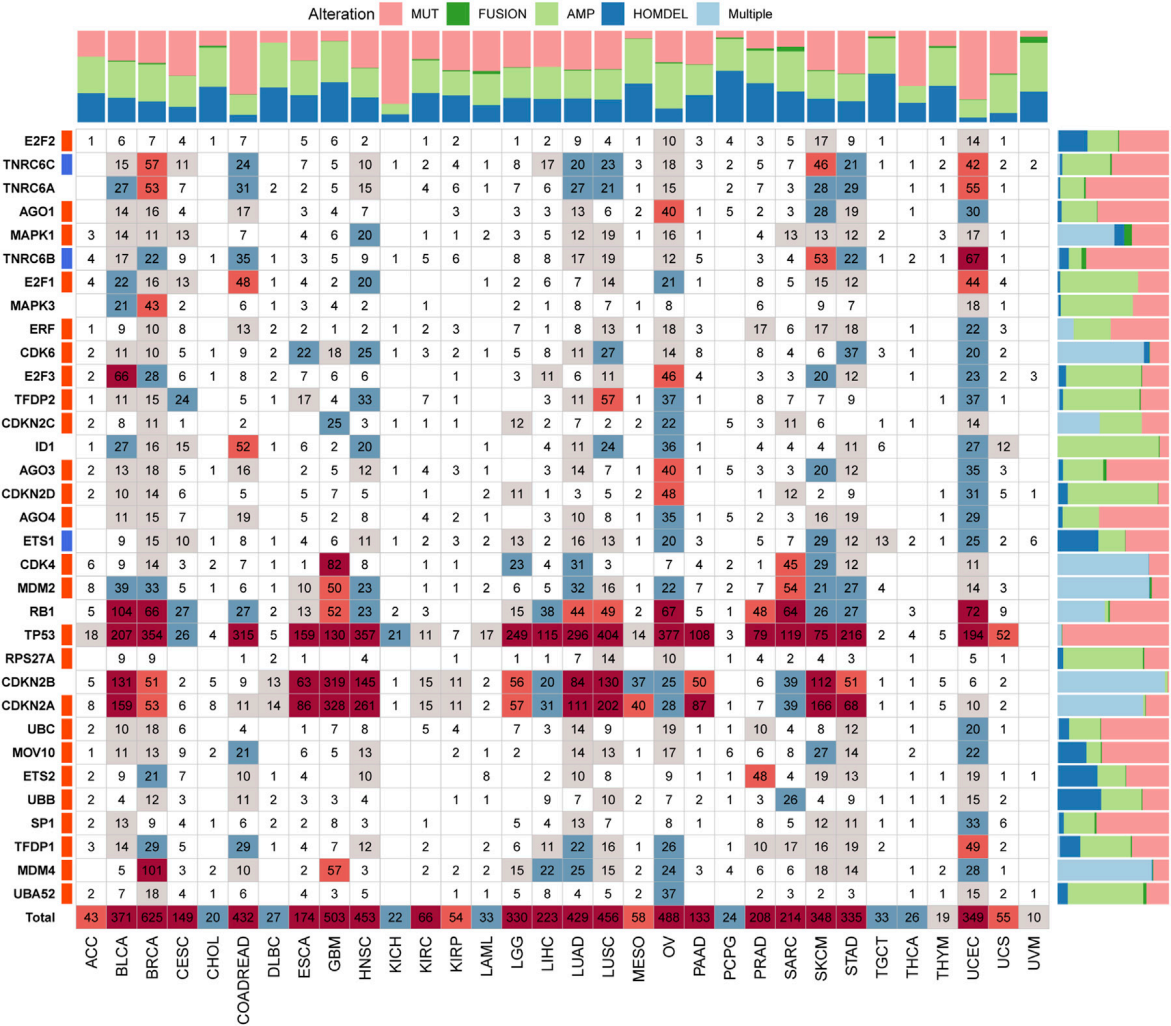
The genetic alteration landscape of senescence-related genes (SRGs) in various cancers. In the pan-cancer, the genetic alterations of SRGs include mutations, fusions, amplifications, deletions, and multiple variations. Numbers in the boxes represent sample sizes for genetic variants; the darker the color, the greater the number. Quantified data were plotted in bar graphs (MUT: mutation; AMP: amplification; HOMDEL: homozygous deletion; and Multiple: multiple variations).

To comprehensively understand the SRGs’ molecular characteristics, we analyzed the genetic variation data using the cBioPortal database. Among all cancers, BRCA had the highest alteration numbers and the types of alteration, including amplification, mutation, homozygous deletion, and fusion; the most frequently altered gene in BRCA was TP53 (Figure 5). Among all SRGs, TP53 was the most commonly altered gene, with “mutation” as the main alteration type, followed by CDKNA and CDKN2B, with “multiple alterations” as the main alteration type (Figure 5). Among these alterations, we observed that the most common genetic alteration was amplification, whereas the rarest was fusion (Figure 5).

### Somatic Mutations of Senescence-Related Genes

**FIGURE 6:**
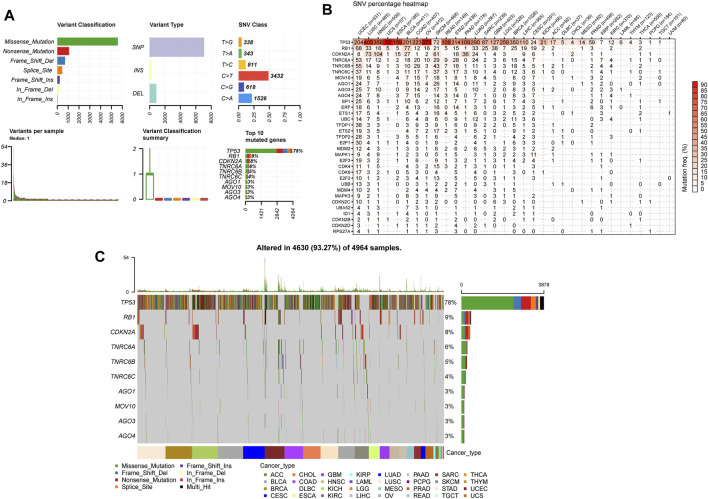
Single nucleotide variation (SNV) analysis in senescence genes. **(A)** The summaries plot of the SNV variants classification of SRGs from the TCGA database. Upper left (variant classification): the count of each deleterious mutation type (Missense_Mutation, Nonsense_Mutation, Frame_Shift_Ins, Frame_Shift_Del, In_Frame_ Del, In_Frame_Ins, and Splice_Site) of SRGs in 33 cancer types. Upper central (Variant Type): the count of single nucleotid polymorphism, INS, and DEL of SRGs in 33 cancer types. Upper right (SNV class): the count of each SNV class of senescence genes in 33 cancer types. Lower left (Variants per sample): the count of variants in each sample. A bar represents a sample, the color of the bar corresponding to the color of variant classification. Lower central (variant classification summary): box plot represents the distribution of the count of each variant classification in the sample set of 33 cancer types. The color of the box corresponds to the color of variant classification. Lower right (Top 10 mutated genes): the count and percentage of variants in top 10 mutated genes. **(B)** Heatmap demonstrating the SNV frequencies of senescence genes across 33 cancer types. For a given cancer, the number of samples with the corresponding mutation gene is indicated by numbers. **(C)** Oncoplot depicting the mutation distribution and the classification of SNV types of top 10 mutated genes from senescence genes in 33 cancer types.

Subsequently, we investigated the SRGs’ single nucleotide polymorphism (SNP) data to detect the frequencies and variant types per tumor subtype. Our analysis of the variant classification revealed that a missense mutation was the most prevalent SNV type in the senescence gene set in TCGA cancer cohorts (Figure 6A). Specifically, the majority of the gene signature mutations included C > T and T > C transversions, followed by C > A transversions (Figure 6A). TP53 was the most frequently mutated among the 33 tumor types among the gene signatures. According to the tumor types, SNVs occurred most frequently by decreasing order in UCEC, LUSC, HNSC, BRCA, LUAD, SKCM, BLCA, ovarian serous cystadenocarcinoma (OV), STAD, and COAD (Figure 6B). Our analysis of the SNV percentage in the gene signatures demonstrated that the top 10 mutated genes were TP53, RB1, CDKN2A, TNRC6A, TNRC6B, TNRC6C, AGO1, MOV10, AGO3, and AGO4, with the mutation frequencies of 78%, 9%, 8%, 6%, 5%, 4%, 3%, 3%, 3%, and 3%, respectively, across TCGA cancer types (Figure 6C). The SNV percentage of SRGs was increased in LUSC, BRCA, HNSC, and OV (Figure 6C). Furthermore, the TP53 mutations highly correlated to the DSS, OS, and PFS of THYM patients. Other genes also demonstrated significant correlations with survival prognosis across various cancer types (Supplementary Table S4).

### Copy-Number Variation Contributes to the Dysregulation of the Senescence-Related Genes

**FIGURE 7:**
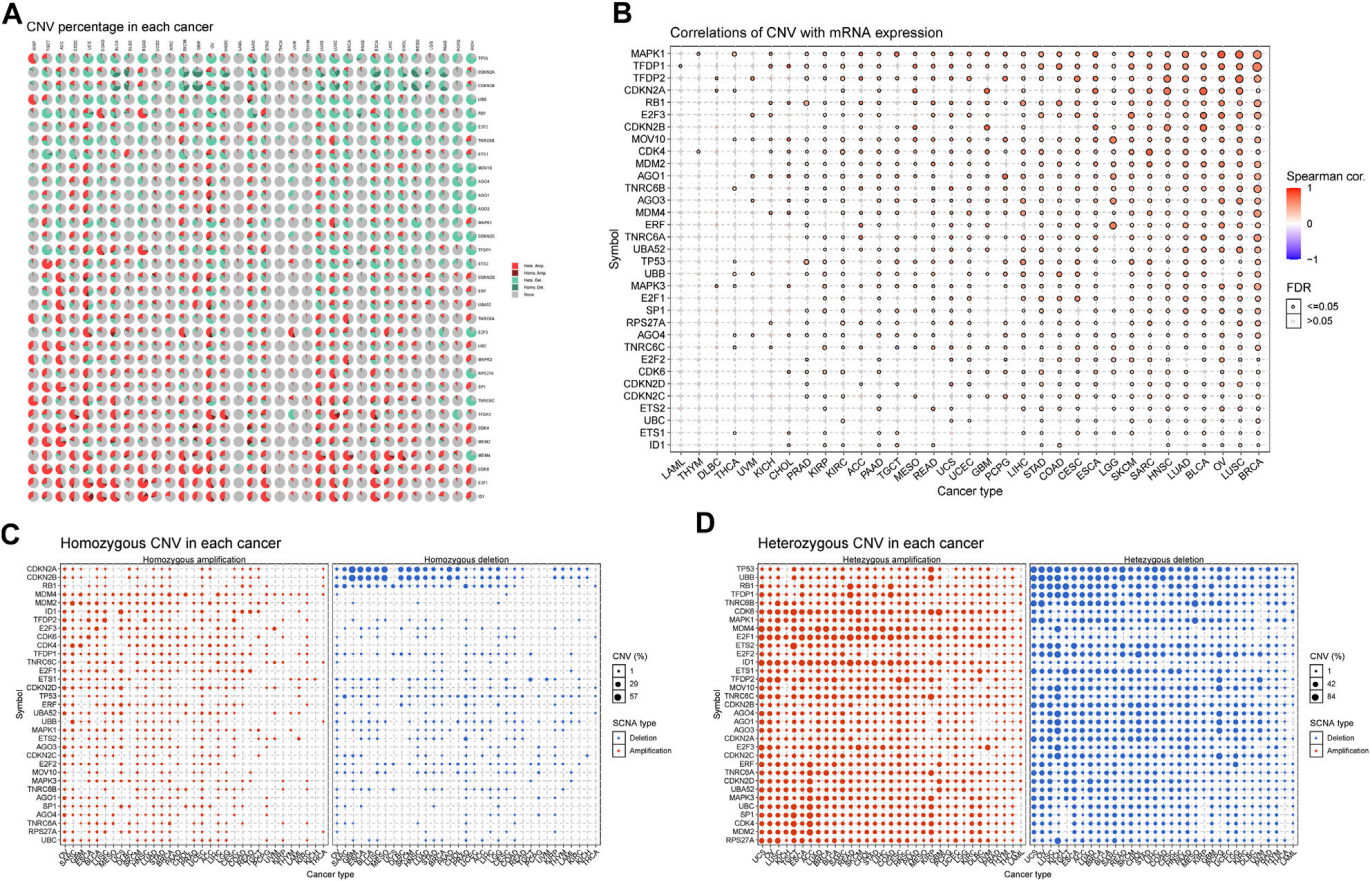
Copy number variation (CNV) analysis of senescence genes. **(A)** Distribution of CNV in 33 cancers. The pie chart summarizes the heterozygous and homozygous CNV of senescence-related genes (SRGs) in 33 cancer types. Each pie chart shows the relative proportion of different mutation types of CNV and different colors indicate different types of CNV. **(B)** CNV relationship with mRNA expression. Bubble plot illustrates the profile between senescence genes’ mRNA expression and CNV level. **(C,D)** Homozygous/Heterozygous CNV profile shows the percentage of homozygous/heterozygous CNVs, including the percentage of deletion and amplification of homozygous/heterozygous CNVs of senescence genes in each cancer type. Only those genes with CNV >5% in the given cancer type are displayed as a point on the graph (Spearman correlation, the *p*-value of the FDR <0.05 was considered significant. FDR: false discovery rate).

CNVs are widespread and pervasive in human cancers and have been proposed to drive tumorigenesis. The distribution of the CNV pie chart indicated that the main mutation types were heterozygous amplification and deletion (Figure 7A). Correlation analyses revealed that the SRGs’ mRNA levels were significantly positively associated with CNVs, especially MAPK1 in OV; TFDP1 in CHOL; CDKN2A and CDKN2B in GBM; CDK4 in SARC; and CDKN2A in BLCA and MESO (Figure 7B). However, there was a weak negative correlation for TNRC6C in TGCT; TFDP1 in LAML; CDKN2C in prostate adenocarcinoma (PRAD); and CDKN2A in KIRC and THCA. Analysis of CNV percentage showed that the main amplified genes of homozygous were ID1 in UCS; TFDP2 in LUSC; MDM2 and CDK4 in SARC; and E2F3 in BLCA (Figure 7C). The deleted CDKN2A and CDKN2B genes in GBM, MESO ESCA, and BLCA were the most obvious type (Figure 7C). Nearly every gene in each tumor showed heterozygous variation (Figure 7D). Heterozygous amplifications of ETS2 and CDK6 in TGCT; CDK6 in GBM; E2F1 and ID1 in rectum adenocarcinoma (READ); and SP1, CDK4, and MDM2 in ACC were all greater than 70% (Figure 7D). Heterozygous deletions of UBB and TP53 in OV; MOV10, AGO1, AGO3, AGO4, CDKN2C, E2F3, MDM4, TP53, UBB, and E2F2 in KICH; TNRC6B in OV and MESO; E2F2 in CHOL; RB1, ETS1, and TFDP1 in TGCT; TP53 and UBB in UCS; TNRC6C in KICH; MOV10 and CDKN2C in Pheochromocytoma and Paraganglioma (PCPG); and MAPK1 in MESO were also all greater than 70% (Figure 7D). These results implied that the CNVs of SRGs mediated their aberrant expression, which suggested that they may play critical roles in tumorigenesis and development.

### Pan-Cancer Analysis of Methylation of Senescence-Related Genes

**FIGURE 8:**
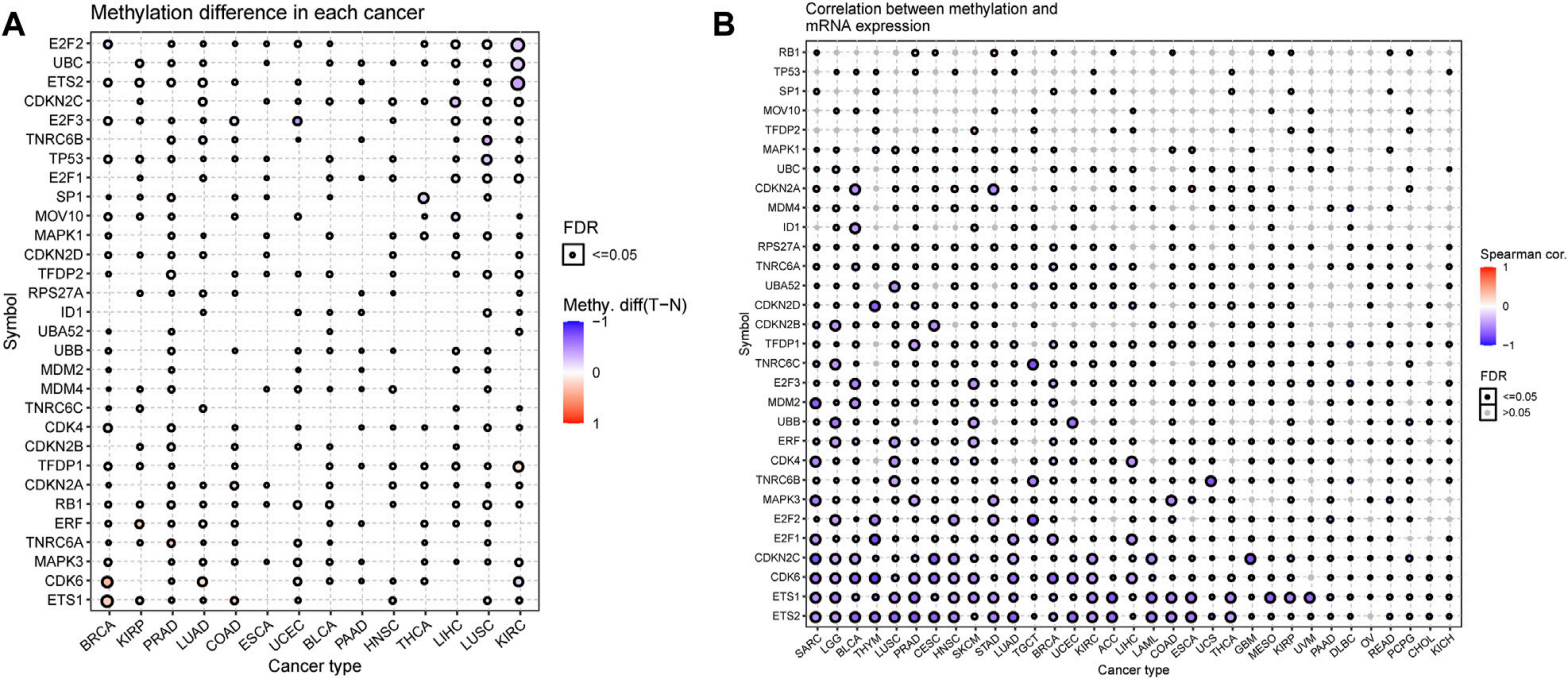
DNA Methylation analysis of senescence-related genes (SRGs). **(A)** Methylation differences of SRGs in tumors. The p-value of the false discovery rate (FDR) and the methylation differences are indicated by the bubble color and bubble size. The bubble color from blue to red represents the methylation difference between tumor and normal. The size dot is positively correlated with FDR significance. **(B)** Correlations of methylation with SRGs’ expression. The blue bubbles represent negative correlations. The red bubbles represent positive correlations; the deeper the color, the higher the correlation. The bubble size is positively correlated with the significance of FDR. The black outline border indicates FDR ≤ 0.05 (Spearman correlation, the dot was filtered by FDR ≤0.05).

Altered DNA methylation has been frequently observed in cancers and is generally known to cause carcinogenesis (Ehrlich, 2002; Rodríguez-Paredes and Esteller, 2011; Koch et al., 2018). Thus, to obtain further insights into the mechanisms affected by the SRGs on tumorigenesis, we also examined the methylation of the senescence gene set to identify epigenetic regulations. We observed that the methylation of SRGs in diverse cancers was found to be highly heterogeneous, and only E2F2 (*n* = 11) and E2F1 (*n* = 9) exhibited hypomethylation in most cancers (Figure 8A). Furthermore, we found that ETS2, TP53, TFDP2, UBB, CDK6, and ETS1 were hypermethylated in 7, 7, 8, 6, 6, and 7 types of cancer, while E2F2, UBC, CDKN2C, E2F3, E2F1, MAPK1, CDKN2D, TFDP1, CDKN2A, and ERF were hypomethylated in 11, 10, 9, 8, 9, 6, 7, 8, 6 and 6 types of cancer (Figure 8A). In addition, the methylation levels of SRGs differed obviously in 14 cancers (Supplementary Table S5). The overall methylation levels and the expression of SRGs were negatively correlated, but CDKN2A in GBM, ESCA, MESO, ACC, PCPG, SARC, UCS, and HNSC; CDKN2B in THYM; ID1 in Diffuse large B-cell lymphoma (DLBC); RB1 in STAD and READ; TFDP2 in SKCM; and MAPK1 in TGCT genes’ expression were significantly positively associated with the levels of methylation (Figure 8B; Supplementary Table S6). These observations indicated that DNA methylation levels might be one of the primary mechanisms regulating the expression of SRGs in cancers.

### Senescence-Associated Biological Pathways

**FIGURE 9:**
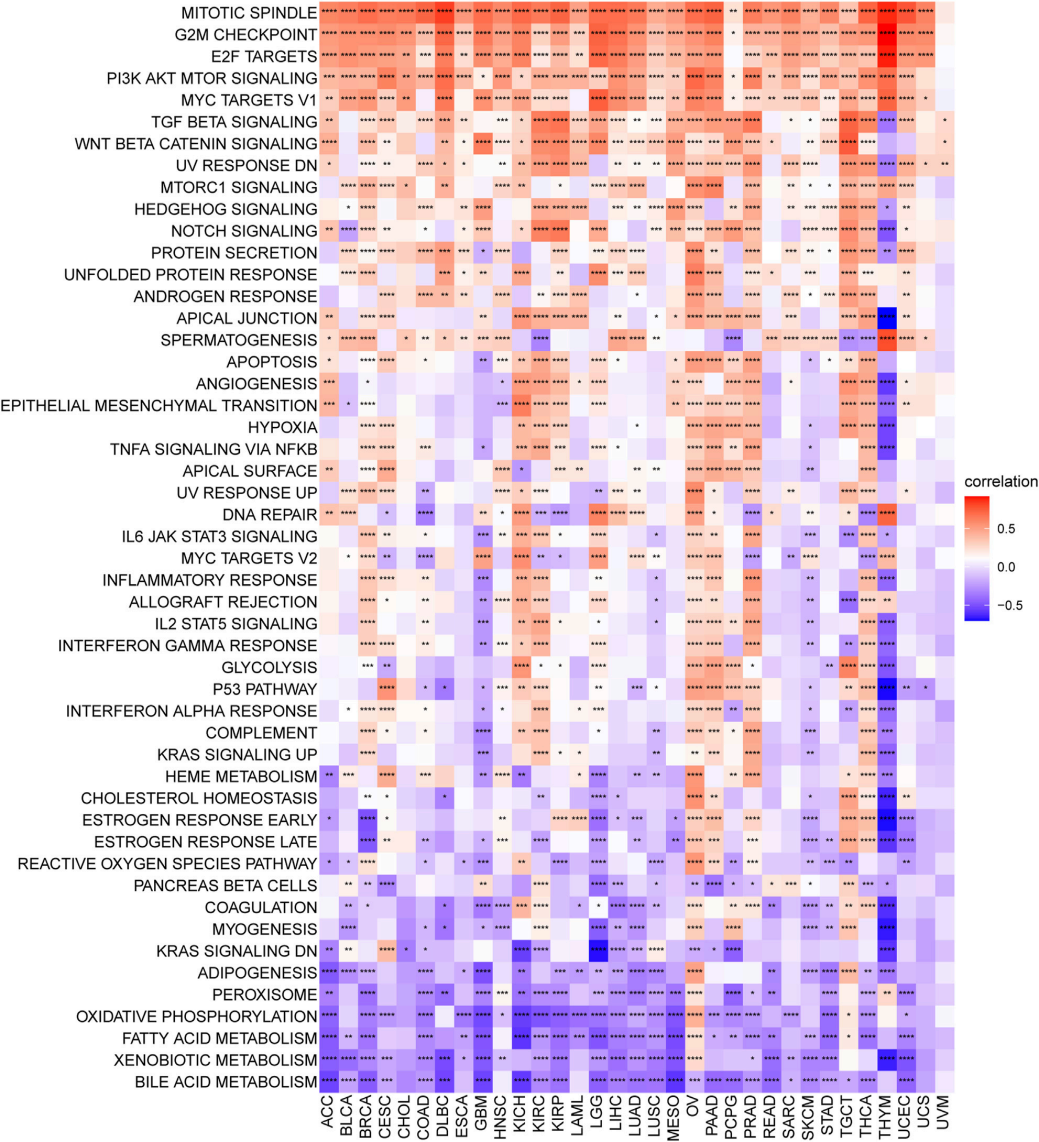
Senescence score correlates hallmark pathways in cancer. Heatmap showing the enrichment of significant hallmarks sets. Each column indicates a tumor type, and each row indicates a hallmark set. Red indicates a positive correlation, and blue indicates a negative correlation. Darker color indicates stronger correlations (Spearman correlation, *p* < 0.05 was considered significant, *p < 0.05, **p < 0.01, ***p < 0.001, and ****p < 0.0001).

Senescence has been demonstrated to affect the regulation of key signaling pathways across multiple levels, eventually giving rise to cancer progression, relapse, and metastasis (Dou and Berger, 2018; Alimirah et al., 2020; Han et al., 2020; Yang et al., 2021). To explore changes in senescence at the pathway/gene-set levels, we implemented the GSVA enrichment score. As a result, cell proliferation-associated signaling pathways, such as mitotic spindle, G2M checkpoint, E2F targets, and PI3K/AKT/mTOR signaling, were found to be positively related to senescence in more than 30 cancer types, confirming that senescence is essential for regulating cell-cycle and tumor growth (Figure 9). Besides, numerous common cancer-related pathways, such as the TGF-beta signaling pathway, Wnt/β-catenin signaling pathway, mTORC1 signaling pathway, Hedgehog signaling pathway, and Notch signaling pathway, were also enriched in multiple cancers with high senescence levels. Various metabolism-related pathways, such as xenobiotic metabolism, adipogenesis, oxidative phosphorylation, bile acid metabolism, peroxisome function, and fatty acid metabolism, were most negatively associated with senescence levels in over 18 (more than half of the total number) tumor types (Figure 9).

### Senescence Score Significantly Correlates With Immune Signatures Among Various Cancers

**FIGURE 10:**
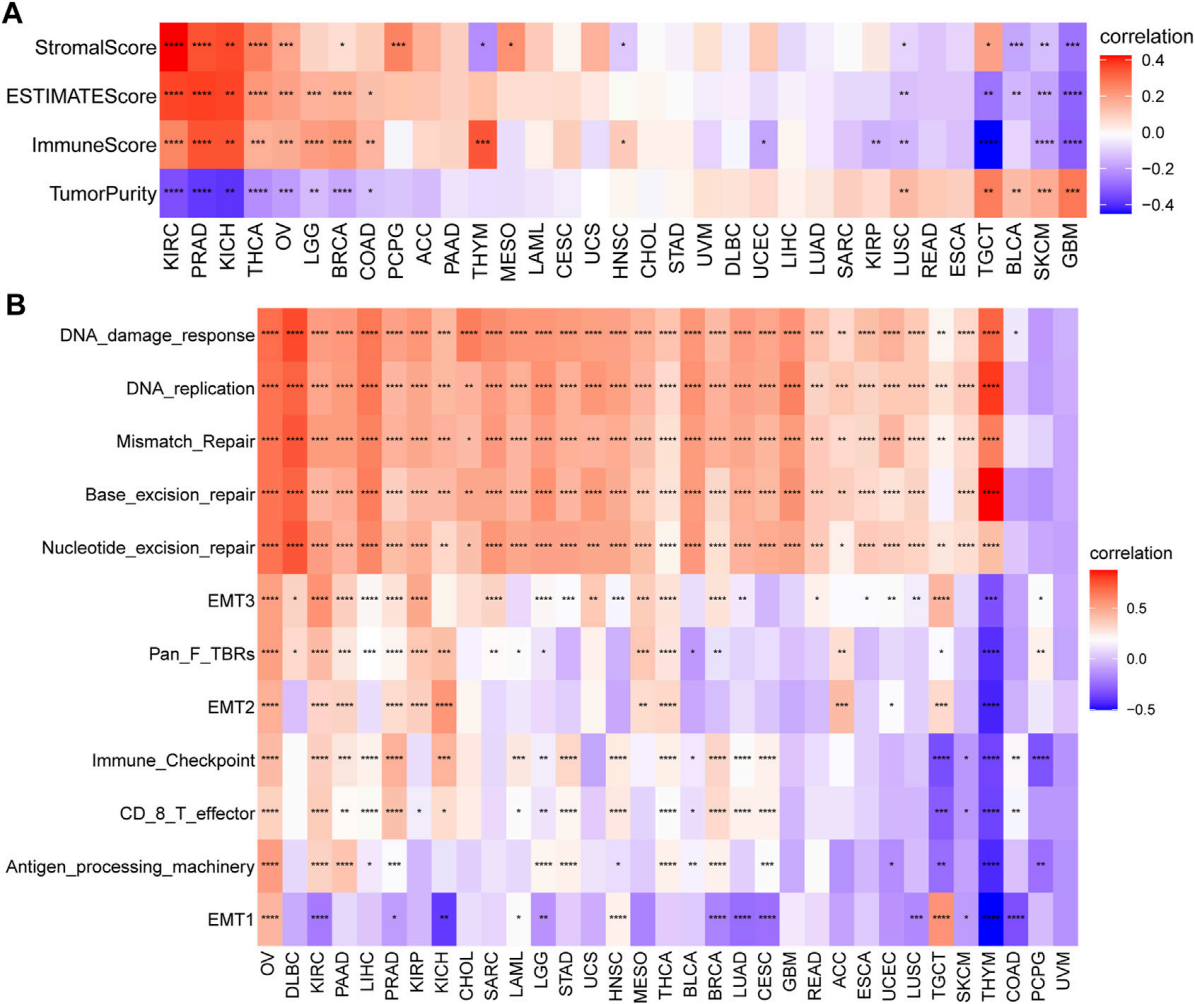
Tumor microenvironment (TME) analysis of senescence score (SS). **(A)** Heatmap indicates the association between SS and TME score in pan-cancer. **(B)** The correlation between SS and biological processes in pan-cancer. Red depicts a positive correlation and blue a depicts negative correlation. Darker color depicts stronger correlations (Spearman correlation, *p* < 0.05 was considered significant, *p < 0.05, **p < 0.01, ***p < 0.001, and ****p < 0.0001).

TME, including cellular and noncellular components, plays a vital role in cancer progression, metastasis, and drug resistance. Therefore, to investigate whether SRGs are involved in the process of immune infiltration in pan-cancer, we first employed the ESTIMATE algorithm to calculate the stromal score, ESTIMATE score, immune score, and tumor purity. The results indicated that, compared with the low-SS group, the high-SS group had a significantly higher ESTIMATE score, immune score, or stromal score in KIRC, PRAD, KICH, THCA, OV, LGG, BRCA, and COAD. However, tumor purity was negatively associated with SS. That is, compared with the low-SS group, the high-SS group had higher immune components but lower tumor purity in the above tumor types (Figure 10A). In contrast, quite the opposite situation was present in SKCM and GBM (Figure 10A). It suggested that senescence was highly involved in immune infiltration and the formation of pluralistic components in multiple cancers. Those findings are consistent with previous observations (Gubin et al., 2014; Alspach et al., 2019; Ruhland and Alspach, 2021). Since accumulating evidence revealed that senescence regulated tumor immunity (Ruhland et al., 2016; He and Sharpless, 2017), we also paid more attention to immune-related and other biological processes (Zeng et al., 2019). In a similar manner, the observations indicated that SS was closely related to immune-related pathways (immune_checkpoint, CD_8_T effector, and antigen_processing _machinery), matrix/metastasis-related pathways (EMTI, EMT2, and EMT3), and DNA damage repair-related pathways (DNA damage response, DNA replication, mismatch repair, base excision repair, and nucleotide excision repair; Figure 10B).

**FIGURE 11:**
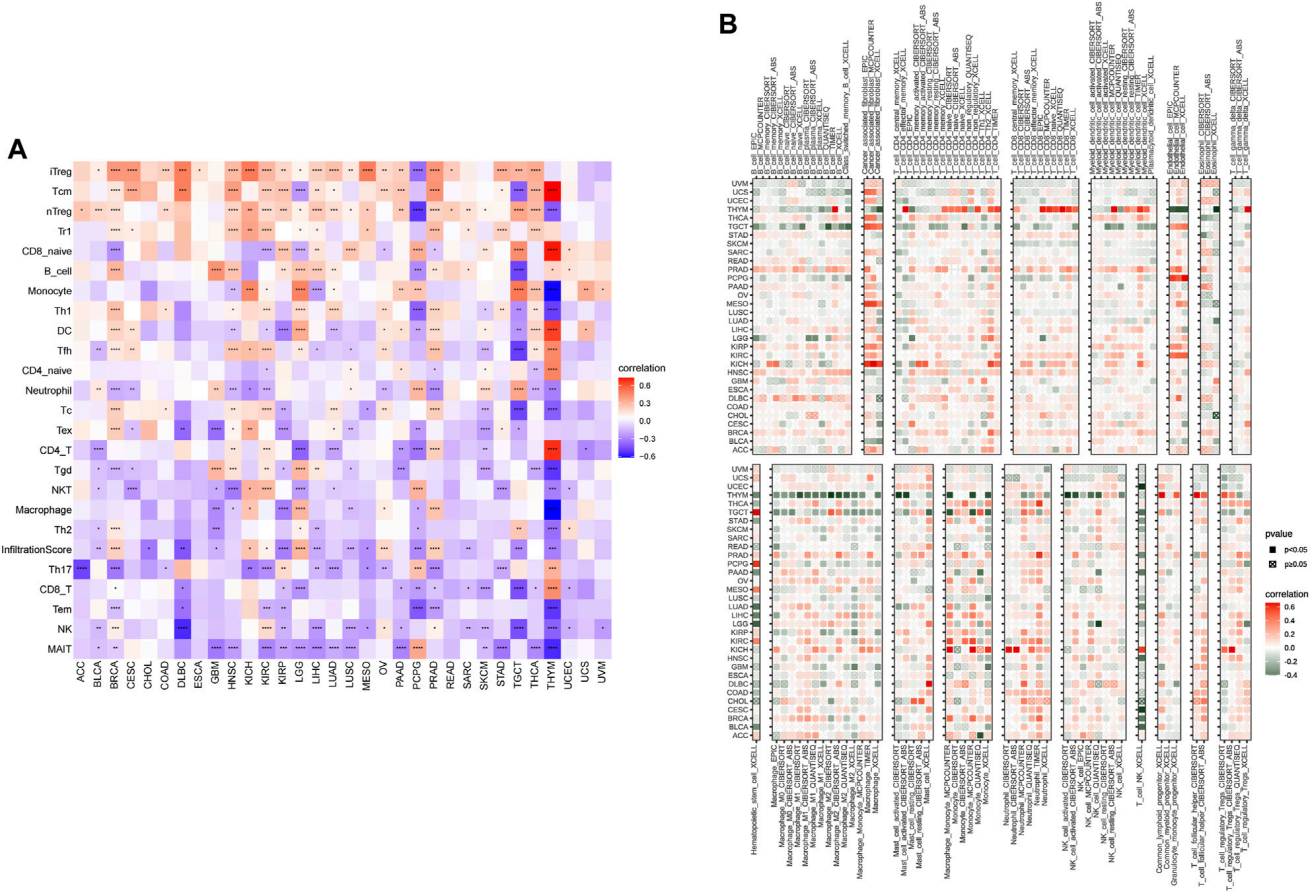
The correlation of senescence score (SS) with the immune cells infiltration. **(A)** Correlation of SS with immune cells infiltration in cancers based on the ImmuCellAI database. **(B)** Correlation of SS with immune cells infiltration in cancers based on the TIMER2 database. Red represents positive correlations and blue represents negative correlations. Darker color represents stronger correlations (Spearman correlation, *p* < 0.05 was considered significant, *p < 0.05, **p < 0.01, ***p < 0.001, and ****p < 0.0001).

To better understand the regulatory role of senescence, we obtained the immune cell infiltration data from different databases to conduct the correlation analyses of immune cell infiltration and SS. We acquired data on 24 immune cells based on the ImmuCellAI database. Overall, the analysis results suggested that the SS was positively associated with multiple infiltrating immune cell populations, such as induced Tregs (iTregs), central memory Ts (Tcms), and natural Tregs (nTregs). In contrast, it was negatively associated with natural killers (NKs) and mucosal-associated invariant Ts (MAITs) in most cancers (Figure 11A). Importantly, we also observed the correlation of SS with TAMs (Figure 11A). Data from the TIMER2 database also supported the above conclusions (Figure 11B). Furthermore, we found a strong positive association of SS with CAFs in some tumors, such as TGCT, MESO, and KICH, from the TIMER2 database (Figure 11B). Besides, CIBERSORT algorithm was applied to analyse the relationships between SRGs and immune cell infiltrates in the tumor microenvironment of each tumor (Supplementary Figure S2). The above data coincide with the suppression of immune surveillance against tumor cells that senescence exerted (Castro et al., 2020).

**FIGURE 12:**
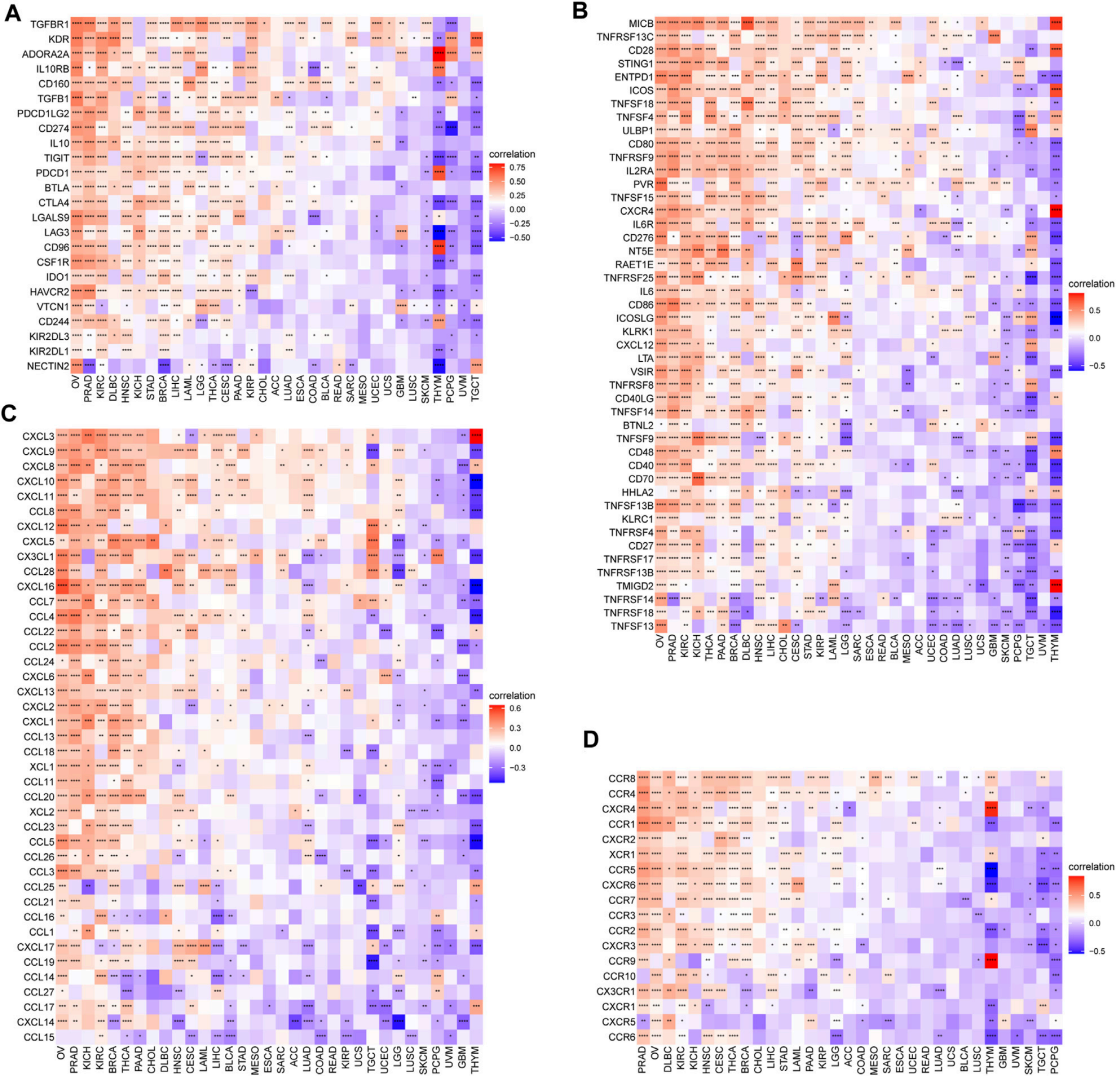
The association between senescence score (SS) and immune-associated genes. **(A)** Correlations between SS and immunosuppressive genes encoding immune suppression. **(B)** Correlations between SS and immune-activated genes encoding immune activation. **(C)** Correlations between SS and chemokines. **(D)** Correlations between SS and chemokine receptors. Red represents a positive correlation and blue represents a negative correlation. Darker color represents stronger correlations (Spearman correlation, *p* < 0.05 was considered significant, *p < 0.05, **p < 0.01, ***p < 0.001, and ****p < 0.0001).

**FIGURE 13:**
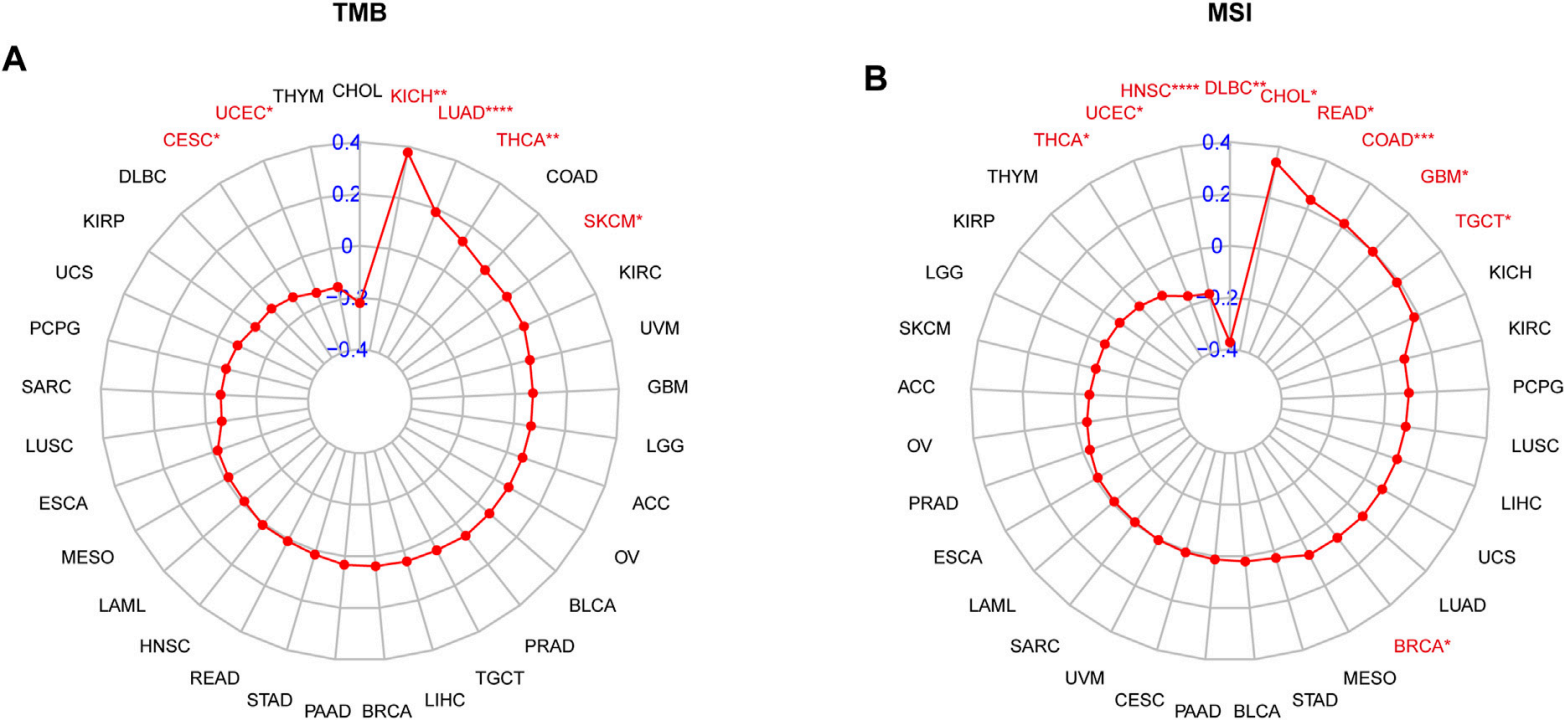
The correlation between senescence score (SS) and immunotherapeutic markers. **(A)** Correlations of SS with TMB. **(B)** Correlations of SS with MSI. The red lines indicate correlation coefficients, and blue values indicate ranges. Red fonts indicate being statistically significant (Spearman correlation, *p* < 0.05 was considered significant, *p < 0.05, **p < 0.01, ***p < 0.001, and ****p < 0.0001).

In addition, we also investigated the associations of SS with immune-associated genes. The study showed that SS was closely linked to immunosuppressive genes (Figure 12A), immune-activated genes (Figure 12B), chemokines (Figure 12C), and chemokine receptors (Figure 12D) in most cancers. It is well known that TMB (Goodman et al., 2017) and MSI (Chalmers et al., 2017) have emerged as predictive biomarkers of improved immunotherapy response across diverse cancers. Therefore, we further investigated their respective associations with senescence levels. We observed that the correlations of SS with TMB achieved significance in six cancers (Figure 13A). Altogether, SS was negatively associated with TMB in UCEC, cervical squamous cell carcinoma, and endocervical adenocarcinoma, while it was positively associated with KICH, LUAD, THCA, and SKCM (Figure 13A). For MSI, the SS exhibited a negative correlation in DLBC, HNSC, UCEC, and THCA but a positive association in CHOL, READ, COAD, GBM, and TGCT (Figure 13B).

### Senescence Score has Prognostic and Predictive Values for Immunotherapy Response

**FIGURE 14:**
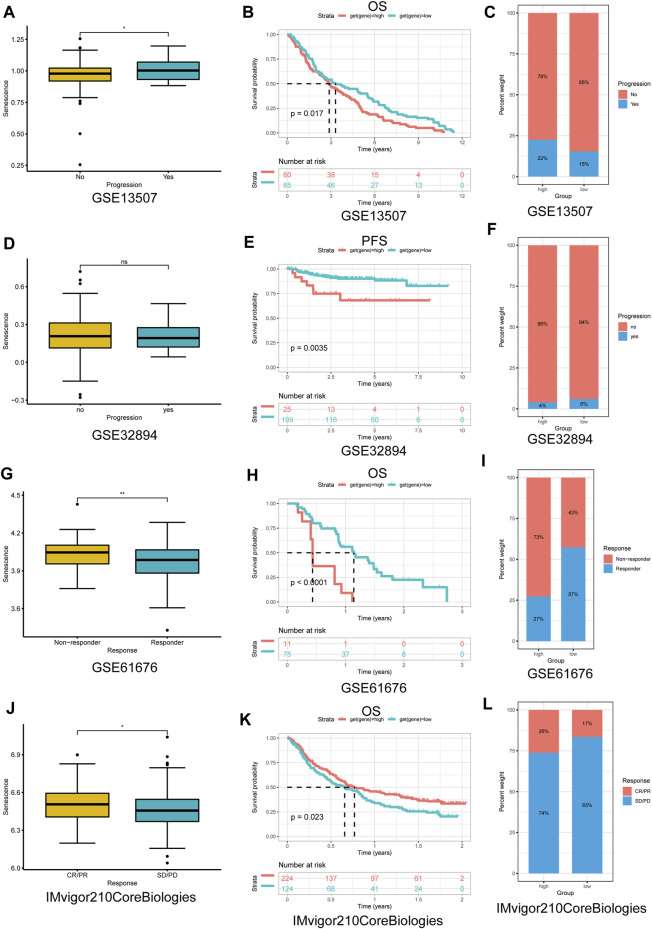
The application of senescence score (SS) in cancer nonimmune/immune-checkpoint therapy. **(A,D,G,J)** The distribution of the SS among samples was stratified by response to nonimmune/immune-checkpoint therapy in each dataset. **(B,E,H,K)** Kaplan–Meier analysis of overall survival/PFS between high- and low-SS groups in each dataset. **(C,F,I,L)** The proportion of response to nonimmune/immune-checkpoint therapy between high- and low-SS groups in each dataset. **(A–I)** Nonimmune-checkpoint therapy; **(J–L)** immune-checkpoint therapy. PFS, progression-free survival; CR, complete response; PR, partial response; SD, stable disease; and PD, progressive disease (*p* < 0.05 was considered significant, *p < 0.05; **p < 0.01).

Immune-checkpoint blockade (ICB) with monoclonal antibodies has emerged as an important anticancer treatment with unprecedented survival benefits (Curran et al., 2010). Because of these observed associations between SS and tumor immunity, we next evaluated the prognostic value of the SS for immunotherapy response by assigning patients in the high- or low-SS cohorts utilizing four associated datasets, including three nonimmune checkpoint therapy datasets (GSE13507, GSE32894, and GSE61676) and one immune-checkpoint therapy dataset from Mariathasan et al. (2018). Compared with the low-SS group, a better prognosis for patients with higher SS had already been described in immune-checkpoint therapy of the IMvigor210CoreBiologies dataset (Figures 14J–L), whereas for other cancer treatment modalities, compared with the low-SS group, patients with higher SS tended to have a poorer prognosis (Figures 14A–I). Together, these results demonstrated that senescence could be a predictive biomarker for response to immunotherapy. Of course, the correlation between senescence and immunotherapy response revealed in this research must be validated clinically.

### Identification of Potential Drugs Targeting Senescence-Related Genes

Finally, we examined the correlation between the SRG expression and the sensitivity of patients to chemotherapy treatment. Interestingly, in accordance with the results of Pearson’s correlation analysis, the high expression of most SRGs (e.g., AGO1, E2F1, CDKN2D, CDKN2A, E2F2, CDKN2C, AGO3, TFDP1, UBA52, TNRC6C, TNRC6B, TNRC6A, E2F3, TP53, TFDP2, and MDM4) was resistant to several GDSC small molecules (positive correlation with IC50) (Supplementary Figure S3A), while it was sensitive to the CTRP small molecules (negative correlation with IC50) (Supplementary Figure S3B). Additional results of the two datasets are presented in Supplementary Tables S7 and S8. These results showed that senescence might mediate resistance to chemotherapy treatment and targeted drug therapy.

## Discussion

Although cellular senescence is traditionally considered a permanent form of cell-cycle arrest that restrains tumorigenesis, a recent study in Nature (Milanovic et al., 2018) pointed out that senescence can counterintuitively promote tumor aggressiveness and cancer sternness. Furthermore, many studies have convincingly demonstrated the paradoxical role of senescence; that is, senescence may be involved in both cancer prevention and cancer aggressiveness (Ruhland et al., 2016; Yang et al., 2021). Thus, we can recognize senescence as a double-edged sword within cancer, demonstrating that it can prevent the occurrence of tumors or, conversely, promote the development of cancer in certain types of malignant tumors. Therefore, given the role of senescence in tumorigenesis and cancer evolution, it is extremely important to investigate senescence in diverse cancer types. Nevertheless, systematic pan-cancer analysis of the role of senescence in diverse cancers is still lacking. Therefore, we conducted a comprehensive characterization of the SRGs across 33 cancers from multiple cancer datasets. We also analyzed the senescence levels in cancers utilizing GSVA and determined the associations between the SS and survival of patients, immune infiltrations, immunotherapy, and drug resistance.

Differential expression analyses showed the tumor context and stage-dependent heterogeneity of SS and the significant differential expression of SRGs across different cancers. Compared with paired normal tissues, increased SS in cancer tissues was observed in 13 cancers, namely BLCA, CHOL, COAD, ESCA, HNSC, KIRC, KIRP, LIHC, LUAD, LUSC, STAD, THCA, and UCEC. In addition, there was a significantly increasing SS with increasing tumor stage in KIRP, KICH, UCEC, and PAAD. The literature supported the above results demonstrating the counter-intuitive tumor-promoting effects on cancer sternness and tumor aggressiveness (Krtolica et al., 2001; Acosta et al., 2008; Dou and Berger, 2018; Milanovic et al., 2018; Yang et al., 2021).

Previous studies reported that senescence plays multifaceted roles (suppressive or progressive effects) in tumorigenesis, depending on the context (Campisi, 2003; Collado et al., 2007). Our univariate Cox regression analysis showed that high SS exhibited a cancer-promoting effect in LGG, ACC, KIRP, PAAD, SKCM, KICH, SARC, MESO, THCA, LIHC, and BRCA patients. In contrast, the opposite effect was found in HNSC, THYM, and GBM patients. Our prognostic analysis suggested that E2F1 was the greatest risk factor among SRGs (Figures 4A,B). This is consistent with the previously reported role of E2F1 in cancers (Chen et al., 2016; Wang et al., 2016; Gnanasundram et al., 2020). Our results confirmed the paradoxical and intricate roles of senescence in tumors.

There is evidence that alterations of genes encoding senescence components often confer susceptibilities to tumors (Rudin et al., 2009; Alam et al., 2016; Augert et al., 2017). In our genetic analysis, BRCA showed the highest alteration numbers compared with other cancers, with “amplification and mutation” as the primary types. Besides, TP53 was the most frequently altered gene, with “mutation” (mainly nonsense mutations) as the primary alteration type, followed by CDKNA and CDKN2B, with “multiple alterations” as the primary alteration type. Interestingly, the patterns of co-occurrences of gene alterations among SRGs were frequently observed across different cancers, which conjoined with the primary genetic driver to promote cancer progression. The above findings might provide new insights into molecular genetic alteration analysis of the SRGs in cancers. Furthermore, the CNV analysis unveiled the high frequencies of copy-number alterations of SRGs, which were positively associated with most of the SRGs’ expression in most tumors, denoting those copy-number alterations could affect SRG expression and, in turn, trigger tumorigenesis.

DNA methylation plays a crucial role in gene expression regulation. Therefore, it has great promise as a noninvasive diagnostic and prognostic biomarker in human cancer (Robertson, 2005; Lawal et al., 2021). Since DNA methylation causes transcriptional silencing (Razin and Cedar, 1991), we predicted that the disrupted growth would be due to hypomethylation in promoter regions leading to several SRGs’ over-expression. Gene methylation regulates gene expression by recruiting repressor proteins or inhibiting the binding of transcription factors to DNA (Moore et al., 2013). However, the positive association between DNA methylation and the expression of SRGs in some cancers indicates the interplay of other regulatory processes other than DNA methylation regulation (Lim et al., 2013; Khan et al., 2015).

In addition, increased senescence was associated with immune-related pathways, DNA damage repair-related pathways, and the activation of several oncogenic processes, such as mitotic spindle, G2M checkpoint, E2F targets PI3K/AKT/mTOR, TGF-beta, and Wnt/beta-catenin signaling pathways. The above results suggested that senescence could mediate the various oncogenic biological processes and serve as an attractive target for cancer therapy. Emerging experimental evidence has also revealed that damaged DNA can accelerate cell senescence and apoptosis and cause cancers (Lv et al., 2019). In addition, numerous studies have reported that senescent cells can acquire characteristics of sternness by activating the Wnt pathway, which can generate tumor-initiating cells (e.g., cancer stem cells) and ultimately cause cancer progression (Milanovic et al., 2018; Muñoz-Galván et al., 2019). Although the senescence-related pathway enrichment analysis uncovered a similar pattern of activating these senescence-related pathways, the pathways, senescence, and cancer interaction networks reflect the high heterogeneity in the susceptibility of diverse tumor types to diverse types of pathway activation.

Cancer immunotherapies by ICB can help the immune system recognize and kill cancer cells. Although immunotherapy offers new hope for treating cancer, disappointingly, only a minority of cancer patients benefit from this immunotherapy (Mahoney et al., 2015; Riley et al., 2019), emphasizing the serious unmet clinical need for identifying the genomic and molecular determinants underpinning immune evasion and the biomarker signature for predicting response to therapy (Jiang et al., 2018). Thus, a better understanding of the immunomodulatory role that senescence plays might help understand the underlying mechanisms of immunoregulation in the TME. It is the entirety of the TME that determines tumor fate. According to the results from the ESTIMATE algorithm, senescence was widely involved in immune infiltration and formation of pluralistic components in KIRC, PRAD, KICH, THCA, OV, LGG, BRCA, and COAD. The further immune infiltration analysis of senescence suggested that SS was positively associated with the infiltration levels of immune suppressive cells, including iTregs, Tcms, and nTregs, and negatively correlated with immune killer cells, such as NKs and MAITs. Previous studies also validated that the senescent environments significantly enhanced the frequency of immunosuppressive regulatory T cells [28] and impacted the innate immune system (Mogilenko et al., 2021). Moreover, we also observed that, in some tumors, senescence was significantly correlated with TAMs and CAFs, which are well known to mediate metastasis (Gaggioli et al., 2007; Gaggioli, 2008; Harney et al., 2015). Furthermore, we further found that senescence was closely linked to immunosuppressive genes, immune-activated genes, chemokines, and chemokine receptors in pan-cancer. These investigations confirm that senescence is closely associated with tumor-immune microenvironments and influences patient prognosis.

Previous research has suggested that patients with a high TMB had better clinical outcomes from immune-checkpoint inhibitors in melanoma (Snyder et al., 2014; Riaz et al., 2017) and urothelial carcinoma (Balar et al., 2017; Snyder et al., 2017). Furthermore, TMB and MSI may serve as useful predictive and prognostic biomarkers for immunotherapy response in human cancers (Li et al., 2020). In the present study, we demonstrated that SS was associated with TMB in six cancer types and with MSI in nine cancer types. Furthermore, compared with the low-SS group, patients with higher SS had a better prognosis in immunotherapy in the IMvigor210CoreBiologies dataset. Our results also suggested the involvement of SRGs in the resistance of human cancer cell lines to small molecule drugs. The role of senescence as a predictor of the prognosis and response to immunotherapy could potentially offer great advancement to cancer treatment.

In conclusion, our systematic pan-cancer analysis indicated that SS exhibited a context-dependent association with cancer prognosis, immune evasion, and therapy response to chemotherapy or immunotherapy. Therefore, senescence may serve as an attractive target for cancer therapy. However, further work will be required to assess the potential of senescence and delineate its precise role in tumorigenesis and the response to therapy.

## Data Availability

The original contributions presented in the study are included in the article/Supplementary Material. further inquiries can be directed to the corresponding author.

## Supplementary Material

Supplementary Figure S1 PPI network of SRGs. The PPI network was constructed using STRING website.

Supplementary Figure S2 Correlation between the 33 SRGs’ expression and immune infiltrating subtypes of 33 cancer types. Blue represents positive correlations and red represents negative correlations (Spearman correlation, *p* < 0.05 was considered significant, **p* < 0.05; ***p* < 0.01; ****p* < 0.001).

Supplementary Figure S3 Correlation of 33 SRGs’ expression with drug sensitivity in cancers. **(A)** Correlation between drug sensitivity of GDSC and SRGs’ expression. **(B)** Correlation between drug sensitivity of CTRP and SRGs’ expression. The negative correlation indicates that the gene’s high expression is sensitive to the drug and vice versa. Blue bubbles represent negative correlations; red bubbles represent positive correlations. The deeper the color, the higher the correlation. Bubble size is positively correlated with the FDR significance. The black outline border indicates FDR ≤0.05.

## References

[B1] Acosta J. C. O'Loghlen A. Banito A. Guijarro M. V. Augert A. Raguz S. (2008). Chemokine Signaling via the CXCR2 Receptor Reinforces Senescence. Cell. 133 (6), 1006–1018. 10.1016/j.cell.2008.03.038 18555777

[B2] Alam S. K. Yadav V. K. Bajaj S. Datta A. Dutta S. K. Bhattacharyya M. (2016). DNA Damage-Induced Ephrin-B2 Reverse Signaling Promotes Chemoresistance and Drives EMT in Colorectal Carcinoma Harboring Mutant P53. Cell. Death Differ. 23 (4), 707–722. 10.1038/cdd.2015.133 26494468 PMC4986638

[B3] Albini A. Bruno A. Noonan D. M. Mortara L. (2018). Contribution to Tumor Angiogenesis from Innate Immune Cells within the Tumor Microenvironment: Implications for Immunotherapy. Front. Immunol. 9, 527. 10.3389/fimmu.2018.00527 29675018 PMC5895776

[B4] Alimirah F. Pulido T. Valdovinos A. Alptekin S. Chang E. Jones E. (2020). Cellular Senescence Promotes Skin Carcinogenesis through p38MAPK and p44/42MAPK Signaling. Cancer Res. 80 (17), 3606–3619. 10.1158/0008-5472.Can-20-0108 32641409 PMC7484313

[B5] Alspach E. Lussier D. M. Miceli A. P. Kizhvatov I. DuPage M. Luoma A. M. (2019). MHC-II Neoantigens Shape Tumour Immunity and Response to Immunotherapy. Nature 574 (7780), 696–701. 10.1038/s41586-019-1671-8 31645760 PMC6858572

[B6] Augert A. Zhang Q. Bates B. Cui M. Wang X. Wildey G. (2017). Small Cell Lung Cancer Exhibits Frequent Inactivating Mutations in the Histone Methyltransferase KMT2D/MLL2 : CALGB 151111 (Alliance). J. Thorac. Oncol. 12 (4), 704–713. 10.1016/j.jtho.2016.12.011 28007623 PMC5669801

[B7] Aunan J. R. Cho W. C. Søreide K. (2017). The Biology of Aging and Cancer: A Brief Overview of Shared and Divergent Molecular Hallmarks. Aging Dis. 8 (5), 628–642. 10.14336/ad.2017.0103 28966806 PMC5614326

[B8] Balar A. V. Galsky M. D. Rosenberg J. E. Powles T. Petrylak D. P. Bellmunt J. (2017). Atezolizumab as First-Line Treatment in Cisplatin-Ineligible Patients with Locally Advanced and Metastatic Urothelial Carcinoma: a Single-Arm, Multicentre, Phase 2 Trial. Lancet 389 (10064), 67–76. 10.1016/s0140-6736(16)32455-2 27939400 PMC5568632

[B9] Balkwill F. R. Capasso M. Hagemann T. (2012). The Tumor Microenvironment at a Glance. J. Cell. Sci. 125 (Pt 23), 5591–5596. 10.1242/jcs.116392 23420197

[B10] Baty F. Joerger M. Früh M. Klingbiel D. Zappa F. Brutsche M. (2017). 24h-gene Variation Effect of Combined Bevacizumab/erlotinib in Advanced Non-squamous Non-small Cell Lung Cancer Using Exon Array Blood Profiling. J. Transl. Med. 15 (1), 66. 10.1186/s12967-017-1174-z 28359318 PMC5372268

[B11] Bonneville R. Krook M. A. Kautto E. A. Miya J. Wing M. R. Chen H.-Z. (20172017). Landscape of Microsatellite Instability across 39 Cancer Types. JCO Precis. Oncol., 1–15. 10.1200/po.17.00073 PMC5972025 29850653

[B12] Campisi J. (2003). Cancer and Ageing: Rival Demons? Nat. Rev. Cancer 3 (5), 339–349. 10.1038/nrc1073 12724732

[B13] Castro A. Pyke R. M. Zhang X. Thompson W. K. Day C.-P. Alexandrov L. B. (2020). Strength of Immune Selection in Tumors Varies with Sex and Age. Nat. Commun. 11 (1), 4128. 10.1038/s41467-020-17981-0 32807809 PMC7431859

[B14] Cerami E. Gao J. Dogrusoz U. Gross B. E. Sumer S. O. Aksoy B. A. (2012). The cBio Cancer Genomics Portal: An Open Platform for Exploring Multidimensional Cancer Genomics Data: Figure 1. Cancer Discov. 2 (5), 401–404. 10.1158/2159-8290.Cd-12-0095 22588877 PMC3956037

[B15] Chalmers Z. R. Connelly C. F. Fabrizio D. Gay L. Ali S. M. Ennis R. (2017). Analysis of 100,000 Human Cancer Genomes Reveals the Landscape of Tumor Mutational Burden. Genome Med. 9 (1), 34. 10.1186/s13073-017-0424-2 28420421 PMC5395719

[B16] Chen C.-L. Uthaya Kumar D. B. Punj V. Xu J. Sher L. Tahara S. M. (2016). NANOG Metabolically Reprograms Tumor-Initiating Stem-like Cells through Tumorigenic Changes in Oxidative Phosphorylation and Fatty Acid Metabolism. Cell. Metab. 23 (1), 206–219. 10.1016/j.cmet.2015.12.004 26724859 PMC4715587

[B17] Collado M. Blasco M. A. Serrano M. (2007). Cellular Senescence in Cancer and Aging. Cell. 130 (2), 223–233. 10.1016/j.cell.2007.07.003 17662938

[B18] Connor A. A. Denroche R. E. Jang G. H. Lemire M. Zhang A. Chan-Seng-Yue M. (2019). Integration of Genomic and Transcriptional Features in Pancreatic Cancer Reveals Increased Cell Cycle Progression in Metastases. Cancer Cell. 35 (2), 267–282.e267. 10.1016/j.ccell.2018.12.010 30686769 PMC6398439

[B19] Curran M. A. Montalvo W. Yagita H. Allison J. P. (2010). PD-1 and CTLA-4 Combination Blockade Expands Infiltrating T Cells and Reduces Regulatory T and Myeloid Cells within B16 Melanoma Tumors. Proc. Natl. Acad. Sci. U.S.A. 107 (9), 4275–4280. 10.1073/pnas.0915174107 20160101 PMC2840093

[B20] Dou Z. Berger S. L. (2018). Senescence Elicits Stemness: A Surprising Mechanism for Cancer Relapse. Cell. Metab. 27 (4), 710–711. 10.1016/j.cmet.2018.03.009 29617638 PMC7205594

[B21] Ehrlich M. (2002). DNA Methylation in Cancer: Too Much, but Also Too Little. Oncogene 21 (35), 5400–5413. 10.1038/sj.onc.1205651 12154403

[B22] Fane M. Weeraratna A. T. (2020). How the Ageing Microenvironment Influences Tumour Progression. Nat. Rev. Cancer 20 (2), 89–106. 10.1038/s41568-019-0222-9 31836838 PMC7377404

[B23] Gaggioli C. (2008). Collective Invasion of Carcinoma Cells. Cell. Adhesion Migr. 2 (1), 45–47. 10.4161/cam.2.1.5705 PMC2635002 19262123

[B24] Gaggioli C. Hooper S. Hidalgo-Carcedo C. Grosse R. Marshall J. F. Harrington K. (2007). Fibroblast-led Collective Invasion of Carcinoma Cells with Differing Roles for RhoGTPases in Leading and Following Cells. Nat. Cell. Biol. 9 (12), 1392–1400. 10.1038/ncb1658 18037882

[B25] Giacomelli A. O. Yang X. Lintner R. E. McFarland J. M. Duby M. Kim J. (2018). Mutational Processes Shape the Landscape of TP53 Mutations in Human Cancer. Nat. Genet. 50 (10), 1381–1387. 10.1038/s41588-018-0204-y 30224644 PMC6168352

[B26] Gnanasundram S. V. Malbert-Colas L. Chen S. Fusée L. Daskalogianni C. Muller P. (2020). MDM2's Dual mRNA Binding Domains Co-ordinate its Oncogenic and Tumour Suppressor Activities. Nucleic Acids Res. 48 (12), 6775–6787. 10.1093/nar/gkaa431 32453417 PMC7337897

[B27] Goodman A. M. Kato S. Bazhenova L. Patel S. P. Frampton G. M. Miller V. (2017). Tumor Mutational Burden as an Independent Predictor of Response to Immunotherapy in Diverse Cancers. Mol. Cancer Ther. 16 (11), 2598–2608. 10.1158/1535-7163.Mct-17-0386 28835386 PMC5670009

[B28] Gubin M. M. Zhang X. Schuster H. Caron E. Ward J. P. Noguchi T. (2014). Checkpoint Blockade Cancer Immunotherapy Targets Tumour-specific Mutant Antigens. Nature 515 (7528), 577–581. 10.1038/nature13988 25428507 PMC4279952

[B29] Han L. Long Q. Li S. Xu Q. Zhang B. Dou X. (2020). Senescent Stromal Cells Promote Cancer Resistance through SIRT1 Loss-Potentiated Overproduction of Small Extracellular Vesicles. Cancer Res. 80 (16), 3383–3398. 10.1158/0008-5472.Can-20-0506 32366480 PMC7611217

[B30] Hänzelmann S. Castelo R. Guinney J. (2013). GSVA: Gene Set Variation Analysis for Microarray and RNA-Seq Data. BMC Bioinforma. 14, 7. 10.1186/1471-2105-14-7 PMC3618321 23323831

[B31] Harney A. S. Arwert E. N. Entenberg D. Wang Y. Guo P. Qian B.-Z. (2015). Real-Time Imaging Reveals Local, Transient Vascular Permeability, and Tumor Cell Intravasation Stimulated by TIE2hi Macrophage-Derived VEGFA. Cancer Discov. 5 (9), 932–943. 10.1158/2159-8290.Cd-15-0012 26269515 PMC4560669

[B32] He S. Sharpless N. E. (2017). Senescence in Health and Disease. Cell. 169 (6), 1000–1011. 10.1016/j.cell.2017.05.015 28575665 PMC5643029

[B33] Huang Y.-H. Hu J. Chen F. Lecomte N. Basnet H. David C. J. (2020). ID1 Mediates Escape from TGFβ Tumor Suppression in Pancreatic Cancer. Cancer Discov. 10 (1), 142–157. 10.1158/2159-8290.Cd-19-0529 31582374 PMC6954299

[B34] Iacobucci I. Li Y. Roberts K. G. Dobson S. M. Kim J. C. Payne-Turner D. (2016). Truncating Erythropoietin Receptor Rearrangements in Acute Lymphoblastic Leukemia. Cancer Cell. 29 (2), 186–200. 10.1016/j.ccell.2015.12.013 26859458 PMC4750652

[B35] Jiang P. Gu S. Pan D. Fu J. Sahu A. Hu X. (2018). Signatures of T Cell Dysfunction and Exclusion Predict Cancer Immunotherapy Response. Nat. Med. 24 (10), 1550–1558. 10.1038/s41591-018-0136-1 30127393 PMC6487502

[B36] Khan S. A. Reddy D. Gupta S. (2015). Global Histone Post-translational Modifications and Cancer: Biomarkers for Diagnosis, Prognosis and Treatment? Wjbc 6 (4), 333–345. 10.4331/wjbc.v6.i4.333 26629316 PMC4657128

[B37] Kim E. Rebecca V. Fedorenko I. V. Messina J. L. Mathew R. Maria-Engler S. S. (2013). Senescent Fibroblasts in Melanoma Initiation and Progression: an Integrated Theoretical, Experimental, and Clinical Approach. Cancer Res. 73 (23), 6874–6885. 10.1158/0008-5472.Can-13-1720 24080279 PMC3926439

[B38] Koch A. Joosten S. C. Feng Z. de Ruijter T. C. Draht M. X. Melotte V. (2018). Author Correction: Analysis of DNA Methylation in Cancer: Location Revisited. Nat. Rev. Clin. Oncol. 15 (7), 467. 10.1038/s41571-018-0028-9 29713045

[B39] Krtolica A. Parrinello S. Lockett S. Desprez P.-Y. Campisi J. (2001). Senescent Fibroblasts Promote Epithelial Cell Growth and Tumorigenesis: a Link between Cancer and Aging. Proc. Natl. Acad. Sci. U.S.A. 98 (21), 12072–12077. 10.1073/pnas.211053698 11593017 PMC59769

[B40] Labidi-Galy S. I. Treilleux I. Goddard-Leon S. Combes J.-D. Blay J.-Y. Ray-Coquard I. (2012). Plasmacytoid Dendritic Cells Infiltrating Ovarian Cancer Are Associated with Poor Prognosis. Oncoimmunology 1 (3), 380–382. 10.4161/onci.18801 22737622 PMC3382863

[B41] Landa I. Pozdeyev N. Korch C. Marlow L. A. Smallridge R. C. Copland J. A. (2019). Comprehensive Genetic Characterization of Human Thyroid Cancer Cell Lines: A Validated Panel for Preclinical Studies. Clin. Cancer Res. 25 (10), 3141–3151. 10.1158/1078-0432.Ccr-18-2953 30737244 PMC6522280

[B42] Lawal B. Lin L.-C. Lee J.-C. Chen J.-H. Bekaii-Saab T. Wu A. (2021). Multi-Omics Data Analysis of Gene Expressions and Alterations, Cancer-Associated Fibroblast and Immune Infiltrations, Reveals the Onco-Immune Prognostic Relevance of STAT3/CDK2/4/6 in Human Malignancies. Cancers 13 (5), 954. 10.3390/cancers13050954 33668805 PMC7956610

[B43] Lawrenson K. Grun B. Benjamin E. Jacobs I. J. Dafou D. Gayther S. A. (2010). Senescent Fibroblasts Promote Neoplastic Transformation of Partially Transformed Ovarian Epithelial Cells in a Three-Dimensional Model of Early Stage Ovarian Cancer. Neoplasia 12 (4), 317–IN3. 10.1593/neo.91948 20360942 PMC2847739

[B44] Lee J.-S. Leem S.-H. Lee S.-Y. Kim S.-C. Park E.-S. Kim S.-B. (2010). Expression Signature of E2F1 and its Associated Genes Predict Superficial to Invasive Progression of Bladder Tumors. Jco 28 (16), 2660–2667. 10.1200/jco.2009.25.0977 20421545

[B45] Li L. Liu X. Sanders K. L. Edwards J. L. Ye J. Si F. (2019). TLR8-Mediated Metabolic Control of Human Treg Function: A Mechanistic Target for Cancer Immunotherapy. Cell. Metab. 29 (1), 103–123.e105. 10.1016/j.cmet.2018.09.020 30344014 PMC7050437

[B46] Li R. Han D. Shi J. Han Y. Tan P. Zhang R. (2020). Choosing Tumor Mutational Burden Wisely for Immunotherapy: A Hard Road to Explore. Biochimica Biophysica Acta (BBA) - Rev. Cancer 1874 (2), 188420. 10.1016/j.bbcan.2020.188420 32828886

[B47] Lim P. S. Li J. Holloway A. F. Rao S. (2013). Epigenetic Regulation of Inducible Gene Expression in the Immune System. Immunology 139 (3), 285–293. 10.1111/imm.12100 23521628 PMC3701174

[B48] Liu C.-J. Hu F.-F. Xia M.-X. Han L. Zhang Q. Guo A.-Y. (2018). GSCALite: a Web Server for Gene Set Cancer Analysis. Bioinformatics 34 (21), 3771–3772. 10.1093/bioinformatics/bty411 29790900

[B49] Liu Q. Liao Q. Zhao Y. (2017). Chemotherapy and Tumor Microenvironment of Pancreatic Cancer. Cancer Cell. Int. 17, 68. 10.1186/s12935-017-0437-3 28694739 PMC5498917

[B50] Lv S. Wen H. Shan X. Li J. Wu Y. Yu X. (2019). Loss of KMT2D Induces Prostate Cancer ROS-Mediated DNA Damage by Suppressing the Enhancer Activity and DNA Binding of Antioxidant Transcription Factor FOXO3. Epigenetics 14 (12), 1194–1208. 10.1080/15592294.2019.1634985 31232159 PMC6791696

[B51] Macosko E. Z. Basu A. Satija R. Nemesh J. Shekhar K. Goldman M. (2015). Highly Parallel Genome-wide Expression Profiling of Individual Cells Using Nanoliter Droplets. Cell. 161 (5), 1202–1214. 10.1016/j.cell.2015.05.002 26000488 PMC4481139

[B52] Mahoney K. M. Rennert P. D. Freeman G. J. (2015). Combination Cancer Immunotherapy and New Immunomodulatory Targets. Nat. Rev. Drug Discov. 14 (8), 561–584. 10.1038/nrd4591 26228759

[B53] Mariathasan S. Turley S. J. Nickles D. Castiglioni A. Yuen K. Wang Y. (2018). TGFβ Attenuates Tumour Response to PD-L1 Blockade by Contributing to Exclusion of T Cells. Nature 554 (7693), 544–548. 10.1038/nature25501 29443960 PMC6028240

[B54] Mayakonda A. Lin D.-C. Assenov Y. Plass C. Koeffler H. P. (2018). Maftools: Efficient and Comprehensive Analysis of Somatic Variants in Cancer. Genome Res. 28 (11), 1747–1756. 10.1101/gr.239244.118 30341162 PMC6211645

[B55] Milanovic M. Fan D. N. Y. Belenki D. Däbritz J. H. M. Zhao Z. Yu Y. (2018). Senescence-associated Reprogramming Promotes Cancer Stemness. Nature 553 (7686), 96–100. 10.1038/nature25167 29258294

[B56] Mogilenko D. A. Shpynov O. Andhey P. S. Arthur L. Swain A. Esaulova E. (2021). Comprehensive Profiling of an Aging Immune System Reveals Clonal GZMK+ CD8+ T Cells as Conserved Hallmark of Inflammaging. Immunity 54 (1), 99–115.e112. 10.1016/j.immuni.2020.11.005 33271118

[B57] Moore L. D. Le T. Fan G. (2013). DNA Methylation and its Basic Function. Neuropsychopharmacol 38 (1), 23–38. 10.1038/npp.2012.112 PMC3521964 22781841

[B58] Munn D. H. Sharma M. D. Johnson T. S. (2018). Treg Destabilization and Reprogramming: Implications for Cancer Immunotherapy. Cancer Res. 78 (18), 5191–5199. 10.1158/0008-5472.Can-18-1351 30181177 PMC6139039

[B59] Muñoz-Galván S. Lucena-Cacace A. Perez M. Otero-Albiol D. Gomez-Cambronero J. Carnero A. (2019). Tumor Cell-Secreted PLD Increases Tumor Stemness by Senescence-Mediated Communication with Microenvironment. Oncogene 38 (8), 1309–1323. 10.1038/s41388-018-0527-2 30305726

[B60] Newman A. M. Liu C. L. Green M. R. Gentles A. J. Feng W. Xu Y. (2015). Robust Enumeration of Cell Subsets from Tissue Expression Profiles. Nat. Methods 12 (5), 453–457. 10.1038/nmeth.3337 25822800 PMC4739640

[B61] Pereira B. I. Devine O. P. Vukmanovic-Stejic M. Chambers E. S. Subramanian P. Patel N. (2019). Senescent Cells Evade Immune Clearance via HLA-E-Mediated NK and CD8+ T Cell Inhibition. Nat. Commun. 10 (1), 2387. 10.1038/s41467-019-10335-5 31160572 PMC6547655

[B62] Razin A. Cedar H. (1991). DNA Methylation and Gene Expression. Microbiol. Rev. 55 (3), 451–458. 10.1128/mr.55.3.451-458.1991 1943996 PMC372829

[B63] Riaz N. Havel J. J. Makarov V. Desrichard A. Urba W. J. Sims J. S. (2017). Tumor and Microenvironment Evolution during Immunotherapy with Nivolumab. Cell. 171 (4), 934–949.e916. 10.1016/j.cell.2017.09.028 29033130 PMC5685550

[B64] Riley R. S. June C. H. Langer R. Mitchell M. J. (2019). Delivery Technologies for Cancer Immunotherapy. Nat. Rev. Drug Discov. 18 (3), 175–196. 10.1038/s41573-018-0006-z 30622344 PMC6410566

[B65] Ritchie M. E. Phipson B. Wu D. Hu Y. Law C. W. Shi W. (2015). Limma Powers Differential Expression Analyses for RNA-Sequencing and Microarray Studies. Nucleic Acids Res. 43 (7), e47. 10.1093/nar/gkv007 25605792 PMC4402510

[B66] Robertson K. D. (2005). DNA Methylation and Human Disease. Nat. Rev. Genet. 6 (8), 597–610. 10.1038/nrg1655 16136652

[B67] Rodríguez-Paredes M. Esteller M. (2011). Cancer Epigenetics Reaches Mainstream Oncology. Nat. Med. 17 (3), 330–339. 10.1038/nm.2305 21386836

[B68] Rudin C. M. Avila-Tang E. Harris C. C. Herman J. G. Hirsch F. R. Pao W. (2009). Lung Cancer in Never Smokers: Molecular Profiles and Therapeutic Implications. Clin. Cancer Res. 15 (18), 5646–5661. 10.1158/1078-0432.Ccr-09-0377 19755392 PMC2950319

[B69] Ruhland M. K. Alspach E. (2021). Senescence and Immunoregulation in the Tumor Microenvironment. Front. Cell. Dev. Biol. 9, 754069. 10.3389/fcell.2021.754069 34692707 PMC8529213

[B70] Ruhland M. K. Loza A. J. Capietto A.-H. Luo X. Knolhoff B. L. Flanagan K. C. (2016). Stromal Senescence Establishes an Immunosuppressive Microenvironment that Drives Tumorigenesis. Nat. Commun. 7, 11762. 10.1038/ncomms11762 27272654 PMC4899869

[B71] Schlattl A. Anders S. Waszak S. M. Huber W. Korbel J. O. (2011). Relating CNVs to Transcriptome Data at Fine Resolution: Assessment of the Effect of Variant Size, Type, and Overlap with Functional Regions. Genome Res. 21 (12), 2004–2013. 10.1101/gr.122614.111 21862627 PMC3227091

[B72] Siegel R. L. Miller K. D. Fuchs H. E. Jemal A. (2021). Cancer Statistics, 2021. CA A Cancer J. Clin. 71 (1), 7–33. 10.3322/caac.21654 33433946

[B73] Sjödahl G. Lauss M. Lövgren K. Chebil G. Gudjonsson S. Veerla S. (2012). A Molecular Taxonomy for Urothelial Carcinoma. Clin. Cancer Res. 18 (12), 3377–3386. 10.1158/1078-0432.Ccr-12-0077-t 22553347

[B74] Snyder A. Makarov V. Merghoub T. Yuan J. Zaretsky J. M. Desrichard A. (2014). Genetic Basis for Clinical Response to CTLA-4 Blockade in Melanoma. N. Engl. J. Med. 371 (23), 2189–2199. 10.1056/NEJMoa1406498 25409260 PMC4315319

[B75] Snyder A. Nathanson T. Funt S. A. Ahuja A. Buros Novik J. Hellmann M. D. (2017). Contribution of Systemic and Somatic Factors to Clinical Response and Resistance to PD-L1 Blockade in Urothelial Cancer: An Exploratory Multi-Omic Analysis. PLoS Med. 14 (5), e1002309. 10.1371/journal.pmed.1002309 28552987 PMC5446110

[B76] Sun Q. Zhang B. Hu Q. Qin Y. Xu W. Liu W. (2018). The Impact of Cancer-Associated Fibroblasts on Major Hallmarks of Pancreatic Cancer. Theranostics 8 (18), 5072–5087. 10.7150/thno.26546 30429887 PMC6217060

[B77] Testoni M. Zucca E. Young K. H. Bertoni F. (2015). Genetic Lesions in Diffuse Large B-Cell Lymphomas. Ann. Oncol. 26 (6), 1069–1080. 10.1093/annonc/mdv019 25605746 PMC4542576

[B78] Toufektchan E. Lejour V. Durand R. Giri N. Draskovic I. Bardot B. (2020). Germline Mutation of MDM4 , a Major P53 Regulator, in a Familial Syndrome of Defective Telomere Maintenance. Sci. Adv. 6 (15), eaay3511. 10.1126/sciadv.aay3511 32300648 PMC7148086

[B79] Tu Q. Hao J. Zhou X. Yan L. Dai H. Sun B. (2018). CDKN2B Deletion Is Essential for Pancreatic Cancer Development Instead of Unmeaningful Co-deletion Due to Juxtaposition to CDKN2A. Oncogene 37 (1), 128–138. 10.1038/onc.2017.316 28892048 PMC5759028

[B80] Wang Y. Alla V. Goody D. Gupta S. K. Spitschak A. Wolkenhauer O. (2016). Epigenetic Factor EPC1 Is a Master Regulator of DNA Damage Response by Interacting with E2F1 to Silence Death and Activate Metastasis-Related Gene Signatures. Nucleic Acids Res. 44 (1), 117–133. 10.1093/nar/gkv885 26350215 PMC4705687

[B81] Xu M. Sizova O. Wang L. Su D.-M. (2017). A Fine-Tune Role of Mir-125a-5p on Foxn1 during Age-Associated Changes in the Thymus. Aging Dis. 8 (3), 277–286. 10.14336/ad.2016.1109 28580184 PMC5440108

[B82] Yang J. Liu M. Hong D. Zeng M. Zhang X. (2021). The Paradoxical Role of Cellular Senescence in Cancer. Front. Cell. Dev. Biol. 9, 722205. 10.3389/fcell.2021.722205 34458273 PMC8388842

[B83] Yoshihara K. Shahmoradgoli M. Martínez E. Vegesna R. Kim H. Torres-Garcia W. (2013). Inferring Tumour Purity and Stromal and Immune Cell Admixture from Expression Data. Nat. Commun. 4, 2612. 10.1038/ncomms3612 24113773 PMC3826632

[B84] Zeng D. Li M. Zhou R. Zhang J. Sun H. Shi M. (2019). Tumor Microenvironment Characterization in Gastric Cancer Identifies Prognostic and Immunotherapeutically Relevant Gene Signatures. Cancer Immunol. Res. 7 (5), 737–750. 10.1158/2326-6066.Cir-18-0436 30842092

