# Supplementary figures and images for: Comprehensive Pan-Cancer Analysis of Senescence With Cancer Prognosis and Immunotherapy
Source: Front Mol Biosci. 2022 Jul 15;9:919274. doi: 10.3389/fmolb.2022.919274 (PMC9334796; doi:10.3389/fmolb.2022.919274)

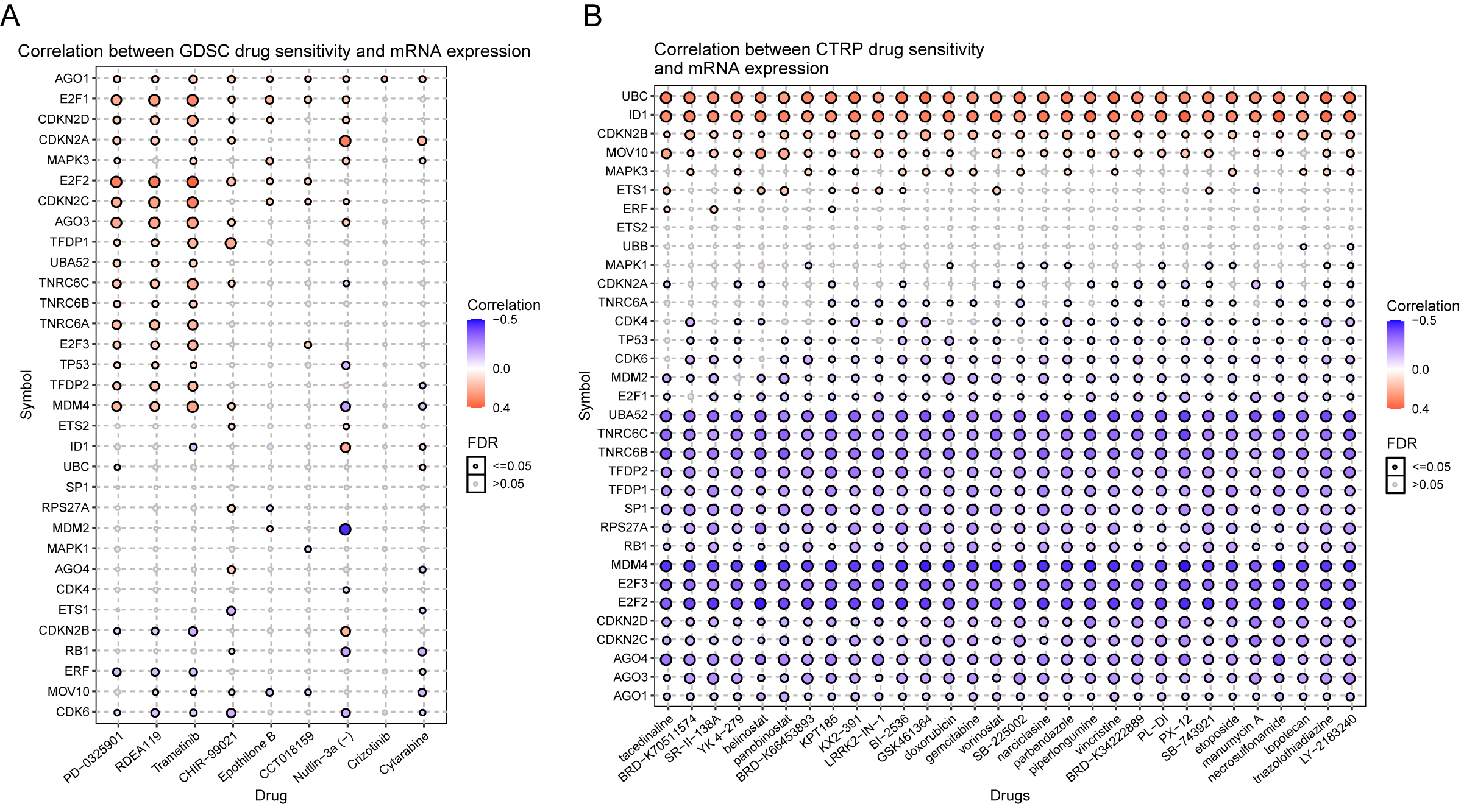

Supplement: Supplementary file 1 [file Image3.TIF]

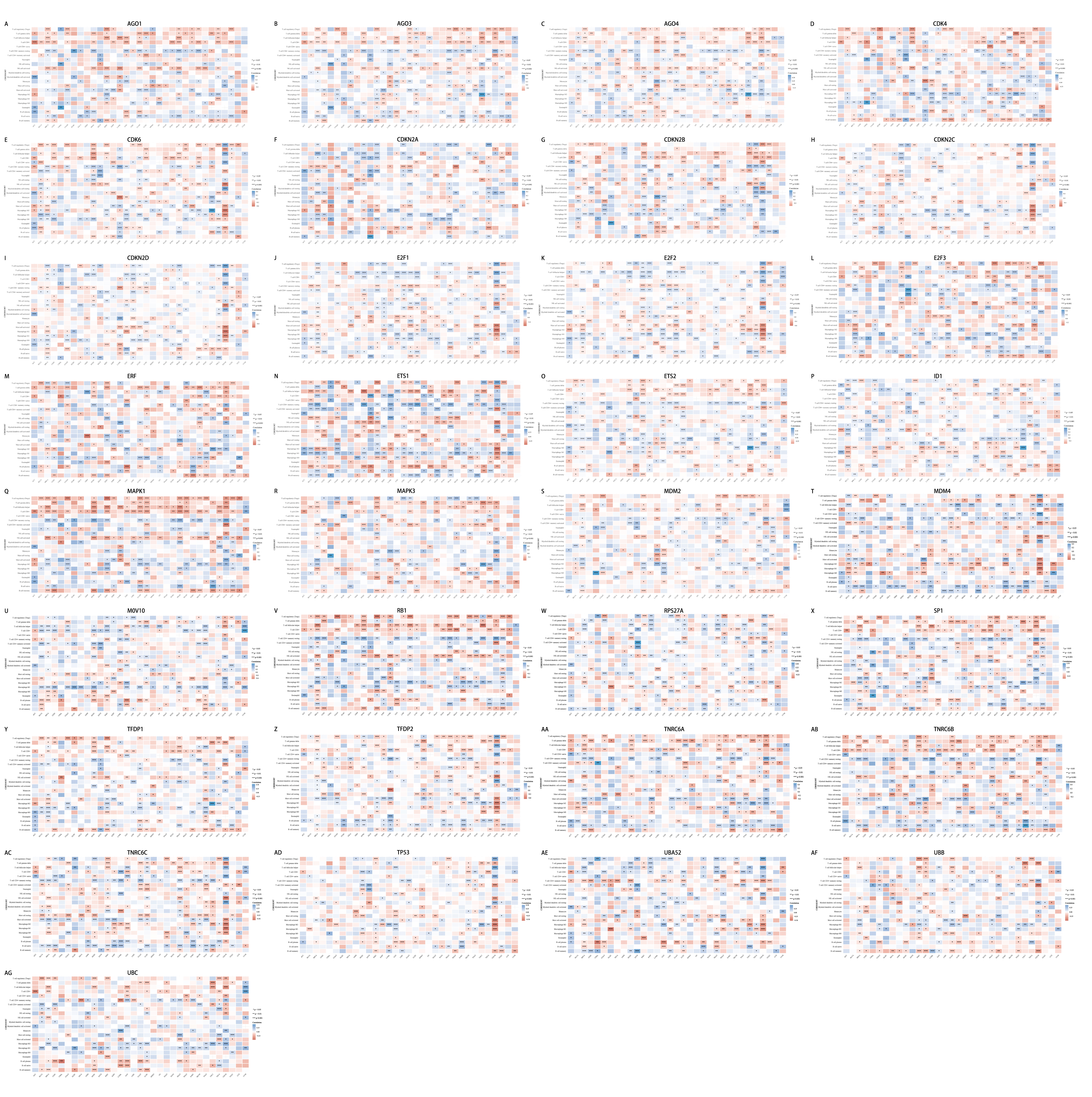

Supplement: Supplementary file 2 [file Image2.TIF]

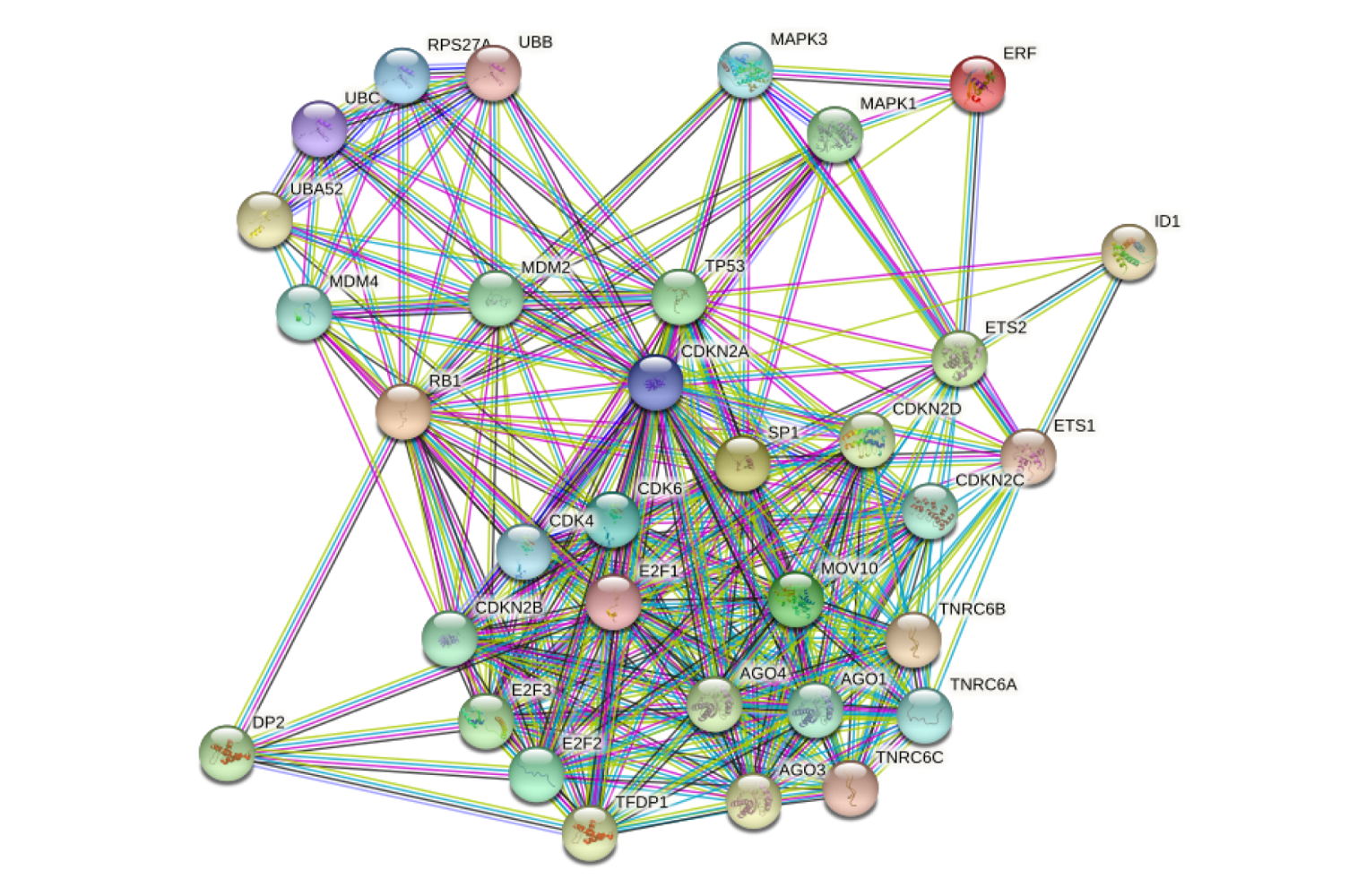

Supplement: Supplementary file 3 [file Image1.TIF]
